# Waste-Tea-Leaf-Derived Microcrystalline Cellulose as a Reinforcement for FDM-Printed PLA: Extraction, Filament Fabrication, Mechanical Performance, and Acoustic Absorption

**DOI:** 10.3390/polym18182293

**Published:** 2026-09-19

**Authors:** Prabhu Cikkuraj, Gokulkumar Sivanantham, Bhuvaneswari Venkateswaran

**Affiliations:** 1 Department of Mechanical Engineering, Christ the King Engineering College, Coimbatore 641104, Tamil Nadu, India; 2 Department of Mechanical Engineering, KPR Institute of Engineering and Technology, Coimbatore 641407, Tamil Nadu, India; gokulkumarmeprof@gmail.com (G.S.); bhuvanashankar82@gmail.com (B.V.)

**Keywords:** microcrystalline cellulose, fused deposition modeling, poly(lactic acid), natural-fiber-reinforced composites, acoustic absorption, mechanical properties, biodegradable composites

## Abstract

Upcycling agro-industrial residues into functional reinforcements supports the circular economy while addressing the brittleness and limited stiffness of poly(lactic acid) (PLA) in fused deposition modeling (FDM). In this study, industrial waste tea leaves were converted into microcrystalline cellulose (WTL-MCC) and used to produce multifunctional PLA composite filaments. WTL-MCC was extracted by dewaxing, alkali treatment, bleaching, and mild acid hydrolysis and characterized by FTIR, XRD, TGA–DTG, DSC, UV–Vis, BET, SEM–EDAX, and AFM. It showed a crystallinity index of 48%, a crystallite size of 2.8 nm and thermal stability to 250 °C (T_max_ = 322 °C). Filaments containing 1, 3, 5, and 7 wt.% WTL-MCC were extruded (1.75 ± 0.05 mm; >97% yield) and printed into ASTM specimens for mechanical (D638/D790/D695) and acoustic (E1050) testing. The properties peaked at 3 wt.% WTL-MCC; beyond this loading, strength declined owing to particle agglomeration and aligned inter-raster voids, confirmed by fractography. The optimized PLA/3MCC composite exhibited mid-to-high-frequency sound absorption (α_max_ = 0.76 at 4000 Hz; NRC 0.385) arising from micro-voids and cellulose surface roughness. Waste-tea-derived MCC, therefore, yields dimensionally consistent printable PLA filaments and multifunctional printed parts, combining an enhanced load-bearing capacity with useful acoustic damping, with 3 wt.% identified as the optimum loading.

## 1. Introduction

Natural-fiber-reinforced composites have attracted steady interest over the past two decades, as industries and individuals have sought alternatives to conventional plastics. Rapid growth in manufacturing, together with rising population and resource demand, has led to increased pollution, greenhouse gas emissions, and solid waste [1]. Conventional plastics are durable, cheap, and easy to shape; however, they remain in the environment for a very long time after use, which has urged material researchers towards biodegradable materials made from renewable sources [2]. The circular economy has become the guiding point behind this shift, treating waste as a raw material rather than a disposal problem and maintaining the value of the material in use for as long as possible [3]. Natural fiber composites are ideal in this regard, as the plant-based reinforcement is renewable, inexpensive, light, biodegradable and less abrasive in processing than glass and carbon fibers [4]. This has led to their widespread use in packaging and automotive applications [5].

Natural fiber sources are abundant, with most being co-products of agriculture and forestry that may be disposed of in landfills or incinerated. Bast fibers, such as flax, hemp, jute, and kenaf; leaf fibers, such as sisal, pineapple leaf, and banana; and wood- or grass-based materials, such as bamboo [6], wood flour [7], and rice husk [8], have all been used to reinforce thermoplastics. Recent studies have examined hemp hurd [9], flax shives [10], wood sawdust [11], pineapple leaf and core [12,13], banana [14], yucca [15], bamboo and jute [16], oat husk [17], hazelnut shells [8], rice husk [18], and forest residues [18]. The composition of these fibers, mainly cellulose, hemicellulose, and lignin, differs with plant type, age, and part used, whether stem, leaf, seed, or grass [4]. This wide choice of low-cost, renewable feedstock is one of the main reasons natural fiber composites continue to grow, but the polymer that binds the fibers together is equally important.

Among the thermoplastics used with natural fibers, poly(lactic acid) (PLA) is the most widely used. It is made from lactic acid produced by fermenting renewable crops, such as corn starch and sugarcane, owing to its bio-based and biodegradable nature [19]. PLA is stiff, clear, and biocompatible, and its low melt viscosity makes it easy to print and is the most common material used in fused deposition modeling (FDM) 3D printing [19]. Neat PLA, however, is brittle, has a low impact strength, has a low heat deflection temperature, has a slow crystallization rate, and is fairly high-cost. Mazur et al. [6] demonstrated that PLA exhibits thermal limitations, as its Young’s modulus decreases by approximately 90% at 80 °C. Consequently, the application of PLA in load-bearing components is restricted, which requires the incorporation of reinforcements. It is crucial that this reinforcement is renewable to maintain the biodegradable characteristics of the material [20].

Most PLA biocomposites use raw or lightly treated fibers and powders; however, purified cellulose is a better form of reinforcement [21]. When cellulose is separated from the fiber structure, it is a long chain of glucose molecules that contain three hydroxyl (-OH) groups that form hydrogen bonds, giving crystalline regions a high stiffness at low densities [4]. The separation of fibers from the cell wall not only removes hemicellulose and lignin, but the reinforcement also becomes more resistant to heat and to humidity since the PLA is melted at high temperatures [22]. The separation process is simply based on chemical methods such as alkali treatment with sodium hydroxide NaOH, which causes swelling of the fiber and removal of surface wax, hemicellulose and part of the lignin. The next step is bleaching with sodium chlorite, NaClO_2_, or hydrogen peroxide, H_2_O_2_, which reduces remaining lignin and color; optionally, a mild acid step is added to cleave remaining non-crystalline segments [23]. These treatments enhance both the purity and crystallinity of cellulose; however, they require careful control to prevent aggressive alkali attacks on cellulose. According to Ceylan et al. [23], a 5% NaOH solution is the most effective treatment for achieving the highest tensile and flexural strengths in PLA. Therefore, maintaining NaOH below 5% is considered safe for maintaining its inherent properties.

Reinforcing purified isolated cellulose into an FDM filament is an effective way to incorporate its benefits into printed parts. The filament is compounded, usually by twin-screw extrusion for good mixing, and then drawn to a steady diameter (usually 1.75 mm) because any change in diameter alters the amount of material fed to the nozzle and leads to uneven parts [24,25]. The addition of a filler increases the melt viscosity and blocks the nozzle. As the plant material consumes water, the feed must be dried well to avoid chain breakage and bubbles. In addition, particle size also plays a vital role, as Xiao et al. [9] showed that increasing the hemp hurd particle size from 35 to 160 µm improved the melt flow but changed the surface finish and strength. Therefore, the required amount of reinforcement must be carefully tuned. Nataraj and Ramesh Babu [26] reported that only 1% horse gram powder raised the tensile, flexural, and compressive strength of PLA by 11.19%, 5.32%, and 5.94%, while 2% and 3% lowered them. Ayyappan et al. [27] measured a 15% rise in tensile strength at 3 vol% *Morinda citrifolia* bark, and Agaliotis et al. [28] found that 1 wt.% henequen flour improved strength and strain but that 2 wt.% and above reduced them. Similarly, Chattrakul et al. [13] linked this improvement to crystallization, measuring an increase in PLA crystallinity from 35.7% to 47.3% with 2 vol% pineapple core powder, with a drop at 3 vol%. Similarly, Matsumoto et al. [29] increased the filament tensile strength and Young’s modulus by 20.0% and 26.6%, respectively, by coating ramie yarn with cellulose nanofibers. Even high loadings can be printed, with up to 20% wood sawdust or forest residues handled without difficulty, although printed parts remain weaker than molded ones because the bonds between layers and the gaps between print paths act as crack starters, making print settings such as raster angle, layer height, and infill density as important as the filler itself [25]. More generally, PLA composites have been designed by FDM for more multifunctional and advanced use cases. For instance, these hierarchically designed 3D-printed PLA/graphene modules have demonstrated combined EMI shielding and thermal management, an illustration of the versatility of the FDM-processed PLA composites [30].

Waste tea leaves are one of the most promising cellulose sources, being one of the most popular beverages in the world [1], and global tea production has already reached 5.07 million tons [4]. Almost all the brewed leaves are disposed of, and due to the water content, they rapidly rot in landfills to produce leachate and methane. Tea bags are also disposed of with the trash [31]. Rahman et al. [4] used data from the Food and Agriculture Organization for the following composition: the raw tea leaf waste fiber amounted to 16.2 wt.% cellulose, 68.2 wt.% hemicellulose, and 18.8 wt.% lignin (due to leaf rather than stem origin). Tea waste in its unprocessed ground form is unsuitable for reinforcement because of its low raw cellulose content. In the same study, alkali treatment raised the cellulose content from 16.2 to 58.8 wt.%, and bleaching raised it further to 87.9 wt.%, while the crystallinity index rose from 41.5% for the raw fiber to 67.3% after alkali treatment, 74.4% after bleaching, and 83.1% after acid hydrolysis, yielding nanocrystals about size of 7.97 nm.

Waste-tea-leaf-derived cellulose can be helpful in achieving both mechanical and acoustic properties. On the mechanical side, a well-dispersed cellulose reinforcement carries the load across a hydrogen-bonded interface, bridges cracks, and helps PLA crystallize; thus, the strength and stiffness increase to an optimum loading before agglomeration reduces them, the same trend observed for other plant fillers [28]. On the acoustic side, sound is absorbed in a porous material when the air moving inside the pores loses energy to friction and heat exchange with the solid, an effect governed by the porosity, tortuosity, airflow resistance, fiber size, and thickness [32]. Cellulose provides additional pores, roughens the inner walls, and provides additional damping to the otherwise rigid and reflective PLA. It is easy to customize the infill, as well as the cell shape and perforations, to optimize the absorption in 3D printing. Zaharia et al. [33] demonstrate a sound absorption coefficient of 0.93 by using PLA filled with ground birch wood at a 40% infill with a triangular pore shape and density, while preserving the compressive and bending strengths at 40 MPa and 50 MPa, respectively. Similarly, Wijekoon et al. [34] reported peak absorption coefficients of 0.83 at 4000 Hz for pineapple leaf fiber and 0.55 at 5000 Hz for water hyacinth fiber at 1 wt.%. Microperforated panels composed of wood–PLA [35,36], cork–PLA [37], and oil palm fiber–PLA [38] adjust the absorption peak by varying the perforation ratio, air gap, and fiber content. Additionally, Bi et al. [39] demonstrated that the voids formed during printing not only constitute defects but also enhance absorption. Panels constructed from waste black tea bags [40] demonstrated a noise reduction coefficient greater than 0.37, highlighting the potential of these materials as reinforcements in the manufacture of 3D printing filaments.

Rahman et al. [4] showed that cellulose and nanocrystals can be obtained from tea leaf waste; however, they only characterized the extracted material and did not prepare a composite. Similarly, Sukthavorn et al. [1] recently mixed waste ground tea leaves directly into PLA at 0–40 phr and, as expected for an untreated filler, found that the tensile strength was lower by 7.12–16.69%, the elongation at break was lower by 12.71–21.35%, and the impact strength was lower by 7.40–33.30%, with only flexural strength and modulus improving by 11.79% and 8.77% at 10 phr, respectively. In addition, Zhao et al. [41] reported the extraction of MCC from Oolong tea waste using chemical treatment and acid hydrolysis, obtaining MCC with a yield of 86.7% and confirming its cellulose I structure and thermal stability. More recently, Debnath et al. [42] developed MCC from black-tea-processing waste using microwave-assisted delignification and bleaching, reporting a crystallinity of 89.77% and an average particle size of approximately 23.06 μm. Tea-waste-derived MCC has also been investigated as a filler in PVA packaging films, demonstrating improvements in mechanical and barrier properties. In the context of additive manufacturing, these studies proved that WTL-filled PLA can be printed, but they also showed the limitations of using waste in its raw form and did not consider acoustic behavior. To the best of our knowledge, no study has extracted cellulose from waste tea leaves, converted it into an FDM filament, or measured both the mechanical and acoustic performance of the printed composite in a single piece of work.

The present study was designed to fill this gap by extracting cellulose from waste tea leaves using a controlled alkali and bleaching process, which was characterized using Fourier transform infrared spectroscopy (FTIR), X-ray diffraction (XRD), scanning electron microscopy (SEM), thermogravimetric analysis (TGA), and Brunauer–Emmett–Teller (BET) surface area analysis to determine the purity, crystallinity, thermal stability, and surface area of the reinforcement. It was then compounded with PLA by single-screw extrusion into filaments of a controlled diameter (1.75 mm) and printed using FDM. The printed composites were tested for mechanical, acoustic, and morphological behaviors in the same study.

The specific objectives of this study were as follows: (i) to extract cellulose from waste tea leaves and study its purity, crystallinity, morphology, surface area, and thermal stability; (ii) to prepare PLA/tea-leaf-cellulose-based composite filaments containing 1, 3, 5, and 7 wt.% cellulose by single-screw extrusion and to check their diameter consistency and printability; (iii) to measure the tensile, flexural, and compressive properties of the FDM-printed composites; (iv) to study the fracture behavior by SEM; (v) to measure the sound absorption coefficient and noise reduction coefficient by the impedance tube method; and (vi) to identify the best cellulose loading.

## 2. Materials and Methods

### 2.1. Materials

Waste tea leaves (WTLs) were collected from Thiashola Tea Estate, Nilgiris, Tamil Nadu, India, from one processing batch generated during conventional tea manufacturing. The processing sequence involved withering, orthodox rolling, oxidation (fermentation), drying (firing), sorting, and grading. The collected WTLs represent the residual tea leaf material generated during tea processing. Commercial poly(lactic acid) (PLA) of grade NatureWorks Ingeo 4043D (NatureWorks, Plymouth, MN, USA), was procured through an authorized distributor in Coimbatore, India. The reported properties of Ingeo 4043D are a density of 1.24 g/cm^3^, melt flow rate of 6–8 g/10 min (210 °C, 2.16 kg), melting point of 145–160 °C, and glass transition temperature of 55–60 °C. Sodium hydroxide (NaOH), sodium chlorite (NaClO_2_), glacial acetic acid (CH_3_COOH), hydrogen peroxide (H_2_O_2_), hydrochloric acid (HCl), and ethanol were of analytical grade and supplied by Priyadarishini Chemicals, Coimbatore, Tamil Nadu, India. Distilled water was used throughout, and all chemicals were used as received without further purification.

### 2.2. Preparation of Waste Tea Leaf Microcrystalline Cellulose (WTL-MCC)

**Figure 1:**
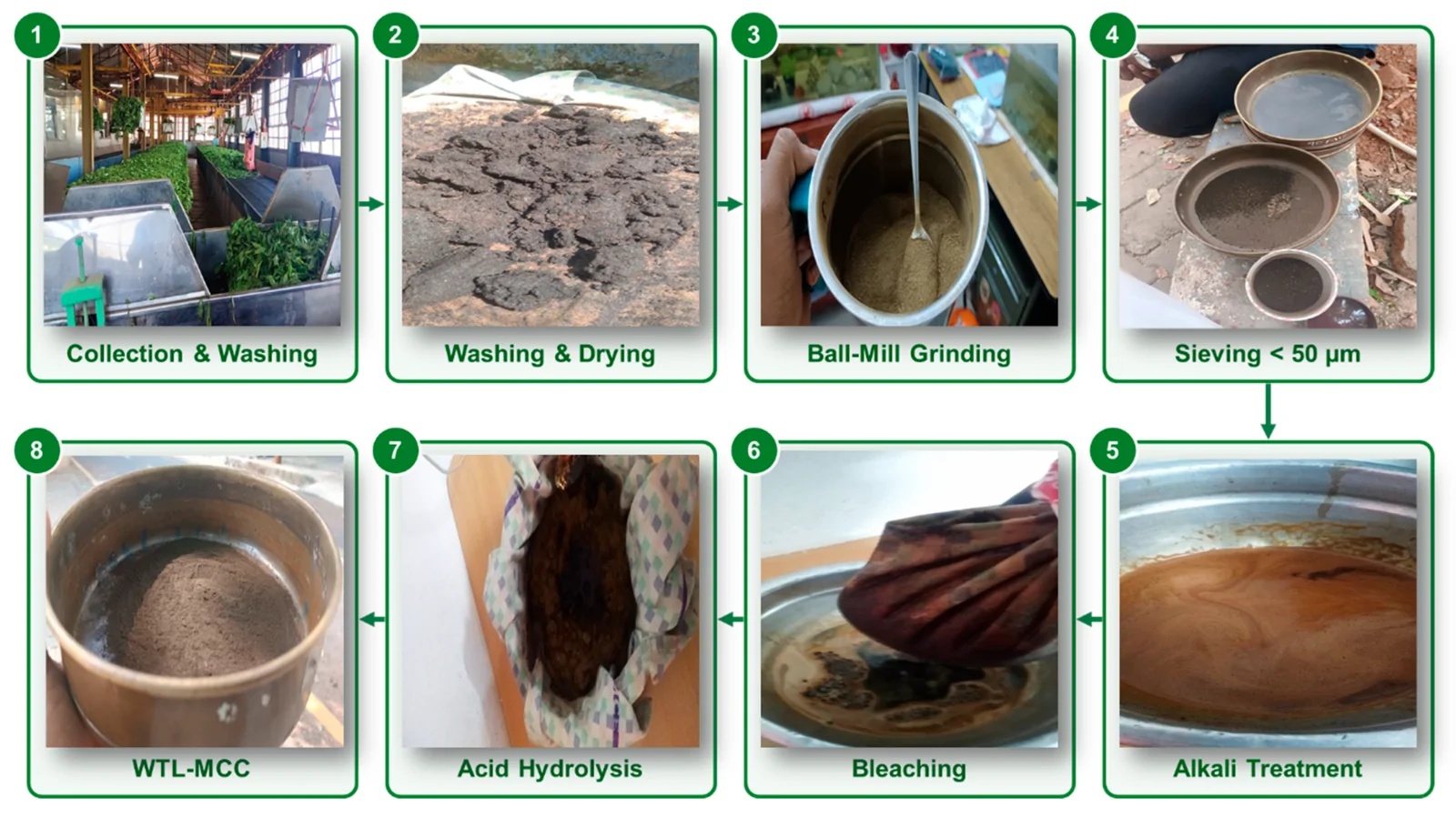
Preparation of waste tea leaf microcrystalline cellulose (WTL-MCC).

Microcrystalline cellulose (MCC) was extracted from waste tea leaves following alkali–bleaching–acid hydrolysis adapted from established procedures for lignocellulosic agro-waste, as shown in Figure 1. The collected leaves were washed several times with distilled water to remove residual tannins, soluble sugars, and dirt, dried in a hot-air oven at 60 °C for 24 h, ground using a planetary ball mill grinder (NST-2A; Tencan, Changsha, China), and sieved to obtain a uniform powder below 50 µm. The powder was dewaxed with a 2:1 (*v*/*v*) toluene–ethanol mixture in a Soxhlet apparatus (Borosil Scientific Ltd., Mumbai, Maharashtra, India) for 6 h and air-dried. The dried powder was first subjected to alkali treatment with 5 wt.% NaOH solution at 80 °C for 2–3 h under continuous stirring to remove hemicellulose, surface waxes and part of the lignin. The residue was filtered and washed with distilled water to neutral pH. Bleaching was performed using 1.7 wt.% NaClO_2_ solution buffered to pH 4–5 with glacial acetic acid at 70–80 °C for 1–2 h, and the step was repeated twice until WTL-MCC was obtained, removing the remaining lignin and colored phenolic compounds. MCC was obtained by a final controlled acid hydrolysis with 2.5 N HCl at 45 °C for 1 h, which selectively solubilized the amorphous cellulose, leaving the crystalline areas intact. The suspension was diluted with cold distilled water, and the acid hydrolysis was interrupted; the suspension was washed several times with distilled water by means of centrifugation, until the supernatant pH fell to 6.5–7. The solid obtained was dried at 50 °C, mildly milled and kept in a desiccator.

### 2.3. Characterization of WTL-MCC

The derived WTL-MCC was characterized for its chemical purity, crystalline nature, thermal stability, surface area and morphology, as well as its application as a reinforcement additive for the melt-processed printed filament of PLA 3D printing filaments. The key factors that determine whether the filler is able to survive the melt compounding and FDM process without much thermal degradation are the efficient removal of hemicellulose and lignin, high crystallinity and good thermal stability up to the PLA processing window.

#### 2.3.1. Fourier Transform Infrared Spectroscopy (FTIR)

The chemical compositions and functional groups of pristine WTLs and WTL-MCC were examined by a JASCO FTIR-4X spectrophotometer (JASCO International Co., Ltd., Hachioji, Tokyo, Japan) with an attenuated total reflectance (ATR) attachment. The powders were pre-dried to reduce the effects of moisture before the spectra were recorded. A few drops of powder were carefully placed on the crystal, and then their FTIR spectra were recorded with data acquisition between 4000 and 400 cm^−1^, with a resolution of 4 cm-1 and 32 scans. The FTIR spectra of the WTL samples (pristine WTLs and WTL-MCC) were compared to confirm the removal of waxes, dissolution of alkali, bleaching of light-absorbing materials, and hydrolysis of cellulose.

#### 2.3.2. X-Ray Diffraction (XRD)

A Bruker D8 ADVANCE X-ray diffractometer (Bruker AXS, Karlsruhe, Germany) was used to study the crystal structure and phase analysis of pristine waste tea leaves (WTLs) and waste tea leaf microcrystalline cellulose (WTL-MCC). Both samples were uniformly packed into a sample holder, and measurements were conducted using monochromatic Cu Kα radiation (λ = 1.5406 Å) at 40 kV and 30 mA. The scanning range was 5–80° (2θ) with a step size of 0.02° and a scan speed of 2° min^−1^. Phase identification was performed using the DIFFRAC.EVA, Version 8.0 (Bruker AXS, Karlsruhe, Germany) against the International Centre for Diffraction Data (ICDD) database, and the crystallinity index (CrI) was determined from the diffraction pattern using the Segal method, shown in Equation (1) [43].

(1) $$CrI\%=\frac{I_{\left(200\right)}-I_{am}}{I_{\left(200\right)}}\times 100$$

where I_200_ is the maximum intensity of the (200) crystalline peak, and I*_am_* is the intensity of the amorphous scatter. In addition, the diffraction angle was used to determine the crystallinity index using the Scherrer equation (Equation (2)) [44].

(2) $$D=\frac{k\lambda }{\beta \cos\theta }$$

where *D*: mean size of the ordered crystalline domain; *K*: dimensionless shape factor (0.9); *λ*: wavelength of the X-ray beam; *β*: line broadening at the full width at half maximum (FWHM); *θ*: Bragg angle (half of the 2θ peak position).

#### 2.3.3. Thermogravimetric Analysis with Derivative Thermogravimetry (TGA–DTG)

The thermal decomposition of pristine waste tea leaves (WTLs) and waste tea leaf microcrystalline cellulose (WTL-MCC) was examined using a PerkinElmer TGA 4000 analyzer (PerkinElmer, Inc., Shelton, CT, USA). Approximately 10 mg of each sample was heated from 25 °C to 900 °C at a rate of 10 °C/min under a nitrogen atmosphere with a gas flow rate of 25 mL/min to minimize oxidation. The mass loss was recorded to evaluate the thermal degradation as the temperature increased, and a derivative thermogravimetric (DTG) curve was employed to determine the temperature at which the maximum degradation rate occurred for the samples.

#### 2.3.4. Differential Scanning Calorimetry (DSC)

The endothermic and exothermic peaks of the WTL-MCC were studied using the Perkin Elmer DSC 6000 (PerkinElmer, Inc., Shelton, CT, USA). About 5 mg of the WTL-MCC obtained was weighed in a crucible and slowly heated from 30 to 400 °C at a rate of 10 °C/min under a nitrogen atmosphere to exclude oxidative damage. The reference used was an empty aluminum pan.

#### 2.3.5. Ultraviolet–Visible (UV–Vis) Spectroscopy

The optical absorption spectrum of WTL-MCC was studied using a Hitachi UH5300 UV–Visible spectrophotometer (Hitachi High-Tech Corporation, Tokyo, Japan) in the range of 190–1100 nm with a spectral bandwidth of 1 nm. The lamp source of the UV–Visible spectrophotometer was a deuterium lamp in the ultraviolet region and a tungsten halogen lamp in the visible region. The WTL-MCC (0.05 g) was dispersed in 100 mL of distilled water with the help of a sonicator for 20 min to prepare the suspension of WTL-MCC; then, the suspension was kept stirred continuously with a stir bar immediately before sampling to ensure the suspension did not settle during measurements. About 3 mL of the suspension was taken in a clean cuvette with a path length of 1.0 cm in quartz, and distilled water was used as the blank. The UV–Visible absorbance spectra were recorded in the wavelength range of 190–1100 nm at room temperature (25 ± 2 °C). The observed spectra were used to identify the characteristic absorption features of WTL-MCC and verify the absence of major UV-absorbing lignin-derived chromophores.

#### 2.3.6. Brunauer–Emmett–Teller (BET) Surface Area Analysis

The porosity and surface characteristics of WTL-MCC were investigated using a BELSORP MAX II (MicrotracBEL Corp., Osaka, Japan). The samples were degassed at 80 °C for 12 h under vacuum prior to nitrogen adsorption at 77 K. The analysis provided the specific surface area, total pore volume, and pore size distribution, which are important for understanding the filler–matrix interaction and its influence on the sound absorption behavior of the composites.

#### 2.3.7. Scanning Electron Microscopy with Energy-Dispersive X-Ray Analysis (SEM–EDAX)

The surface morphology and microstructure of the WTL-MCC were examined using FESEM (Sigma 300, Carl Zeiss AG, Oberkochen, Germany), which offers a resolution of 1.0 nm at 15 kV and 1.6 nm at 1 kV and supports accelerating voltages from 0.02 to 30 kV with magnification up to 1,000,000×. Prior to imaging, the samples were gold-coated using a sputter coater to improve conductivity and reduce charging, and imaging was performed at an accelerating voltage of 10–15 kV to balance image clarity and sample stability. EDX spectra were used to determine the weight and atomic percentage of each element and evaluate the presence of residual inorganic species originating from the chemical treatment.

#### 2.3.8. Atomic Force Microscopy (AFM)

The surface topography of WTL-MCC was analyzed qualitatively and quantitatively in one- and two-dimensional profiles using an NT-MDT scanning probe microscope (NT-MDT Spectrum Instruments, Moscow, Russia). The NTEGRA-series system provides a scan range of up to 200 × 200 µm in the XY plane and 20 µm in Z, a Z-noise floor as low as 0.05 nm and closed-loop linearity of 0.03%. In this study, a high-resolution scan head (2D/3D) reaching 0.1 nm was used, with a scan area of 10 × 10 µm along the X- and Y-axes and a *Z*-axis range of 70 µm. From the height data, the surface topography and roughness characteristics, namely, the average roughness (R_a_), root-mean-square roughness (R_q_), ten-point average roughness (R_z_), skewness, and kurtosis, were determined.

### 2.4. Preparation of 3D Printing Filament of WTL-MCC/PLA

**Figure 2:**
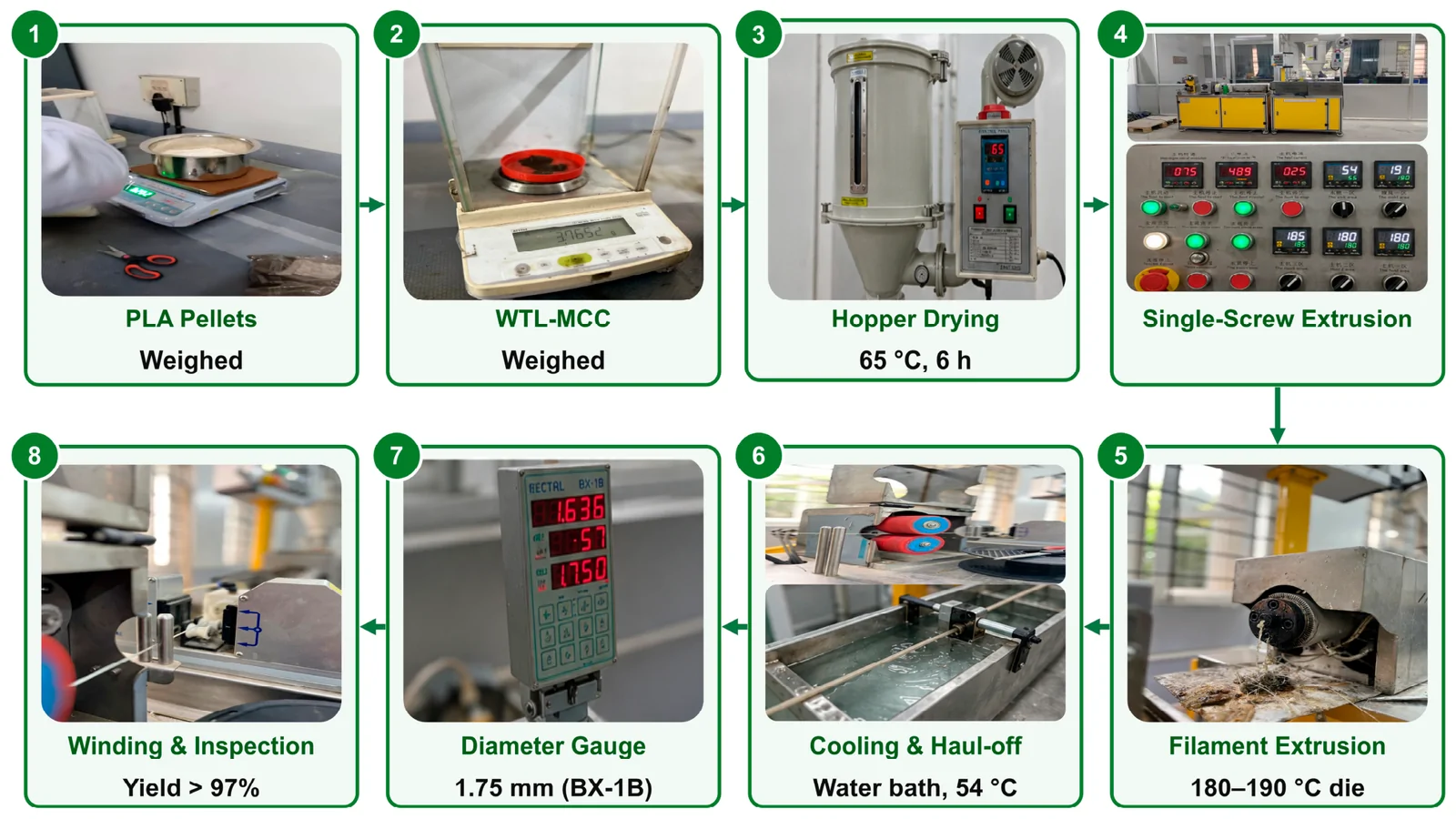
Fabrication of WTL-MCC/PLA filaments.

Prior to filament extrusion, commercial poly(lactic acid) (PLA) pellets and extracted waste tea leaf microcrystalline cellulose (WTL-MCC) were dried separately in a hopper dryer (Model: XMTE-6000, JDYB, Ningbo, China) at 65 °C for 6 h to remove absorbed moisture and minimize hydrolytic degradation during melt processing. The dried PLA pellets and WTL-MCC were subsequently dry-blended according to the compositions listed in Table 1, containing 1, 3, 5, and 7 wt.% WTL-MCC, while neat PLA was used as the control material. The detailed fabrication procedure is illustrated in Figure 2.

Prior to extrusion, the PLA pellets and WTL-MCC were dried separately at 65 °C for 6 h to minimize moisture-related processing defects. The dried samples were weighed according to desired compositions, mixed and then extruded by a FRIEND FLD-25 single-screw filament extruder (Zhangjiagang Friend Machinery, Zhangjiagang, China) with a screw diameter of 25 mm and an L/D ratio of 28:1, with a recommended feeding capacity of 1–5 kg/h. During the extrusion process, the temperature profile was gradually increased from 180 °C (Zone 1) to 180 °C (Zone 2), 185 °C (Zone 3) and 190 °C (die zone) to enable complete melting of the PLA matrix material and uniform distribution of WTL-MCC particles. The extrusion was performed at a screw speed of approximately 40 rpm through a circular die opening with a nominal diameter of 1.75 mm. Subsequently, the extruded filament was fed through a water bath (at 54 °C), followed by a haul-off to facilitate a consistent diameter. The diameter of the filaments was maintained by continuously measuring the diameter using an EECTRL BX-1B online laser diameter gauge (BX-1B; EECTRL, Shanghai, China) and winding onto the collection spools. The processing parameters were optimized to produce continuous filaments with a smooth surface, uniform diameter, without any evident agglomerates, voids, or filament breakage. The WTL-MCC/PLA filaments were visually inspected for surface quality and homogeneity of diameter before 3D printing the test specimens. The fiber diameter was maintained at 1.75 ± 0.05 mm, and total extrusion yield was >97% for all formulations.

### 2.5. Preparation of FDM-Printed WTL-MCC/PLA Composite Test Specimens

**Figure 3:**
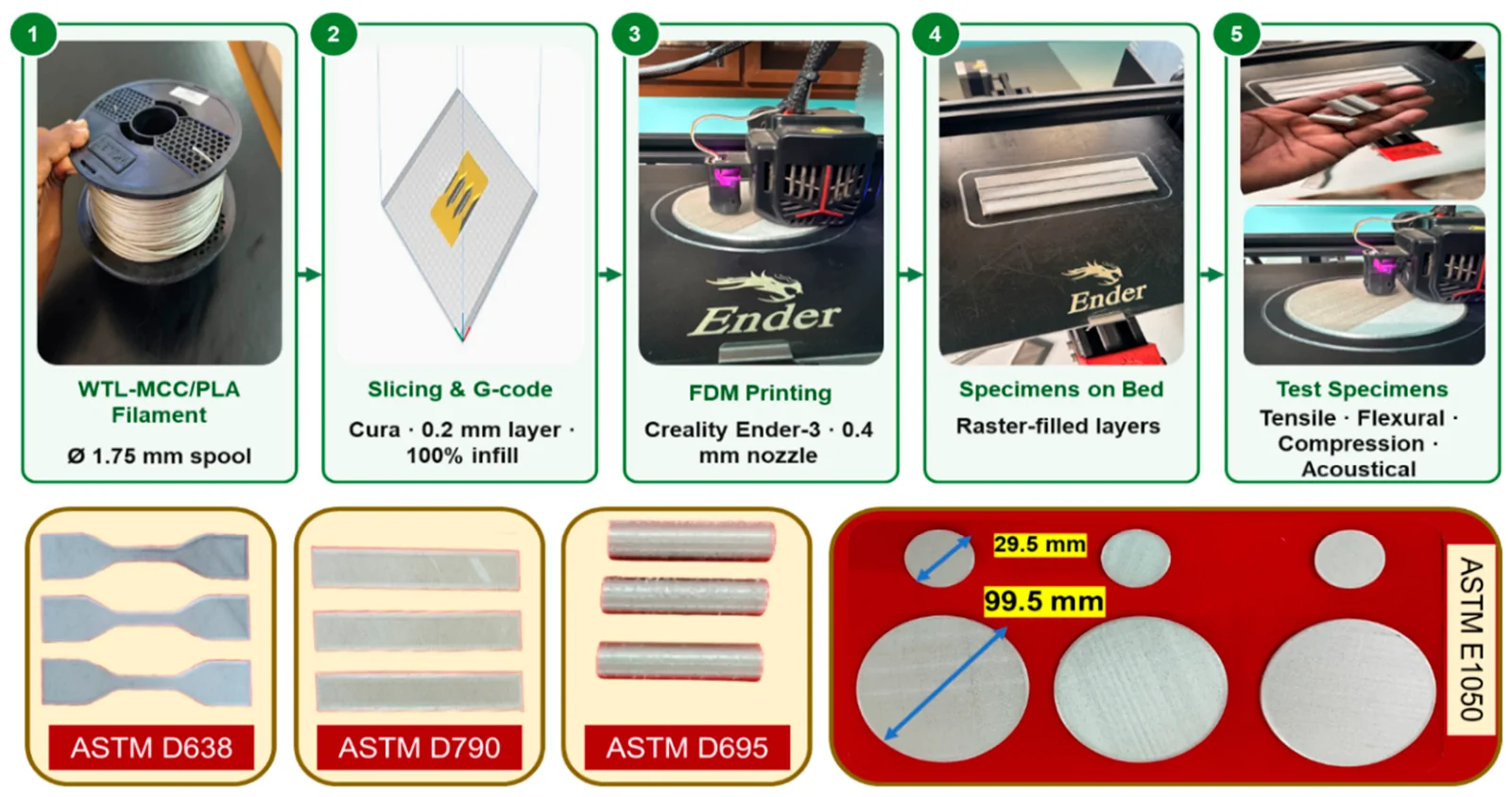
FDM-Printed WTL-MCC/PLA composite test specimens [45,46,47,48].

Test specimens of the prepared WTL-MCC/PLA composite filaments were then fabricated using a fused deposition modeling (FDM) 3D printer (Ender-3, Creality, Shenzhen, China), as shown in Figure 3. 3D printing was performed based on specimen geometries designed in Solid Works 2023 (Dassault Systmes, Vélizy-Villacoublay, France), following the applicable ASTM standards and saved as stereolithography (STL) files. The STL files were then imported into Ultimaker Cura (Version 5.1.0, Ultimaker B.V., Utrecht, The Netherlands), the printing parameters were optimized, and G-code was generated. Standard specimens of various geometries were then successfully 3D-printed to test various mechanical and physical characteristics of the specimens, i.e., tensile, flexural, compression, and acoustic impedance, in conformity with the ASTM D638 (Type I) [45], D790 [46], D695 [47], and E1050 standards [48] respectively.

The printing parameters used were a brass nozzle of 0.4 mm diameter, a nozzle temperature of 215 °C, and a heated build platform temperature of 60 °C. A layer thickness of 0.20 mm was selected as the best compromise for mechanical accuracy and print speed. The specimens were printed with a 100% rectilinear infill density to avoid internal void formation and facilitate consistent mechanical property behavior, with the print and travel speeds fixed at 30 and 60 mm/s, respectively. The optimized FDM printing parameters used to produce each specimen are summarized in Table 2. After fabrication, the specimens were naturally cooled to room temperature on the build platform, then carefully separated from it. Any minor deformation of the edges was removed to the standard specimen dimensions without affecting their shape and structure. The printed specimens were visually examined for any dimensional inaccuracies and manufacturing-related defects such as warping, under-extrusion, layer separation, and voids. Only defect-free specimens satisfying ASTM dimensional requirements were selected for subsequent mechanical and acoustic characterization.

### 2.6. Mechanical Characteristics of 3D-Printed WTL-MCC/PLA Composites

The mechanical performance of the FDM-printed WTL-MCC/PLA composite specimens was evaluated via tensile, flexural, and compression testing using a universal testing machine (Instron 8801; Instron Corporation, Norwood, MA, USA) with a maximum load capacity of 100 kN. All mechanical tests were conducted under ambient laboratory conditions (25 ± 2 °C and 50 ± 5% relative humidity), and a minimum of three replicates were tested for each composition to ensure statistical reliability.

#### 2.6.1. Tensile Testing

The tensile properties of the printed composites were determined in accordance with ASTM D638 (Type I) using an Instron 8801 universal testing machine. The specimens were loaded under displacement-controlled conditions at a crosshead displacement rate of 1.5 mm/min until failure. The maximum tensile strength, Young’s modulus, and elongation at break were calculated automatically using the Bluehill Universal software (Version 4.52) based on the recorded load–displacement data. The fracture behavior of the failed specimens was subsequently used for morphological examination.

#### 2.6.2. Flexural Testing

The flexural behavior of the printed composites was evaluated using a three-point bending configuration following ASTM D790. The tests were performed on an Instron 8801 universal testing machine equipped with a 100 kN load cell. A support span length of 60 mm and crosshead displacement rate of 2 mm/min were used throughout the experiment. The flexural strength and flexural modulus were calculated from the load–deflection curves obtained during testing.

#### 2.6.3. Compression Testing

The compressive properties of the printed composites were measured according to ASTM D695 using an Instron 8801 universal testing machine. Compression loading was applied at a displacement rate of 2 mm/min until specimen failure or significant plastic deformation occurred. The compressive strength and compressive modulus were determined from the stress–strain response generated during the test. The results were compared to evaluate the influence of WTL-MCC loading on the compressive load-bearing capability of the FDM-printed composites.

### 2.7. Acoustical Characteristics of 3D-Printed WTL-MCC/PLA Composites

The acoustic performance of the FDM-printed WTL-MCC/PLA composites was investigated using an Impedance Tube Kit (Type 4206, Brüel & Kjær, Nærum, Denmark) following the transfer function method specified in ASTM E1050–12. The system provided an effective frequency range of 50 Hz–6.4 kHz. Circular specimens with diameters of 99.5 mm and 29.5 mm were prepared to match the corresponding impedance-tube diameters and securely mounted in the sample holder. The specimens were tightly fitted to prevent peripheral air leakage, with no intentional air gap between the specimen and rigid backing, and no additional sealing material was applied around the specimen perimeter. Three independent measurements were performed for acoustic evaluation, and the sound absorption coefficient values were expressed as mean ± standard deviation. The normal-incidence sound absorption coefficient (α) was determined from the measured transfer function between the two microphones, and the noise reduction coefficient (NRC) was calculated as the arithmetic mean of the absorption coefficients at 250, 500, 1000, and 2000 Hz.

### 2.8. Morphological Studies of 3D-Printed WTL-MCC/PLA Composites

The fractured surfaces of the tensile-tested WTL-MCC/PLA composite specimens were examined using field-emission scanning electron microscopy (FESEM) coupled with energy-dispersive X-ray analysis (SEM–EDAX) (Sigma 300, Carl Zeiss AG, Oberkochen, Germany) to investigate the fracture mechanisms, filler dispersion, and fiber–matrix interfacial characteristics. The fractured specimens were sputter-coated with 20nm gold coating prior to imaging to improve their electrical conductivity and mitigate surface charging. Micrographs were taken using 10–15 kV at several different magnifications to observe the fracture surface morphology, layer adhesion, filament fusion, interlayer bonding, voids, crack growth, filler agglomeration, particle pullout and deformation of the matrix due to the FDM printing process. The micrographs were accompanied by EDAX tests to analyze the elements present on the fractured surface, examine the distribution of WTL-MCC in the matrix, and rule out the presence of large WTL-MCC particles. The combined SEM–EDAX analysis provided valuable insight into the microstructural features governing the mechanical and acoustical behavior of the developed FDM-printed composites.

## 3. Results and Discussions

### 3.1. Fourier Transform Infrared (FTIR) Spectroscopy

**Figure 4:**
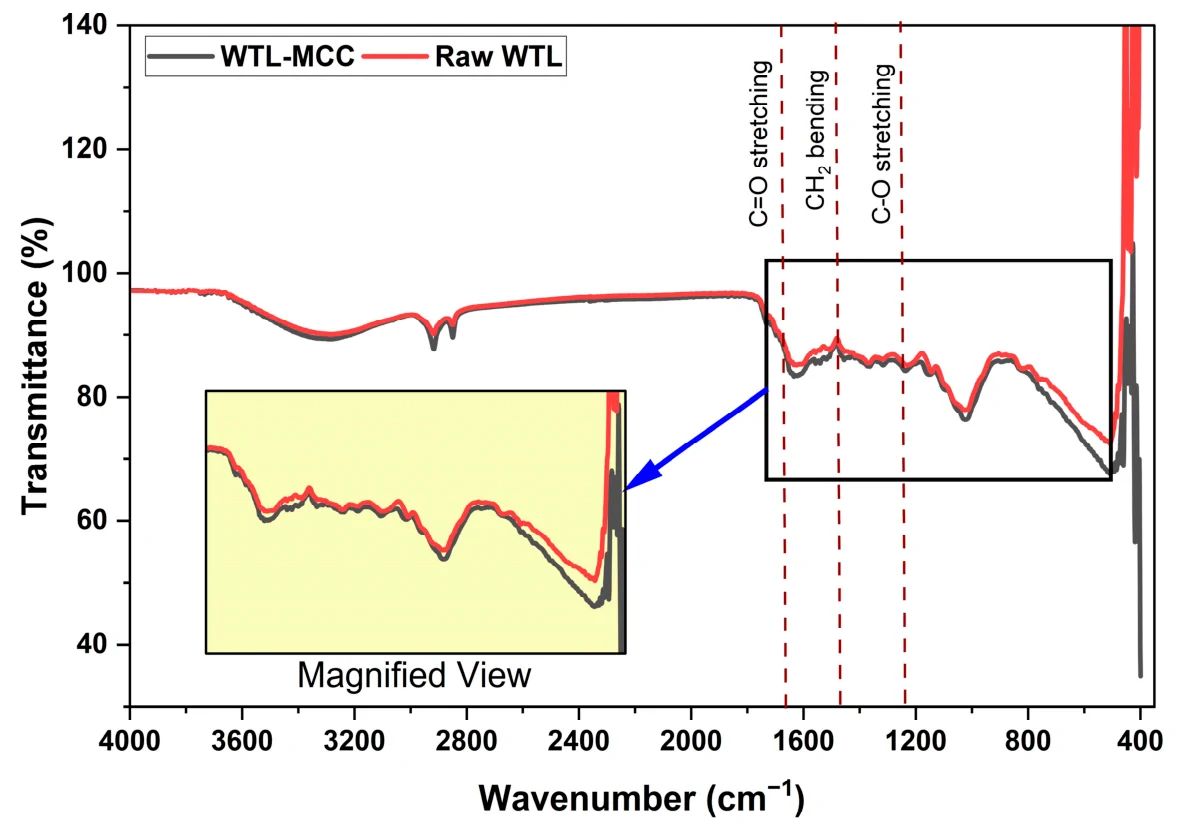
Comparative FTIR spectra of pristine waste tea leaves (WTLs) and waste-tea-leaf-derived microcrystalline cellulose (WTL-MCC).

FTIR spectroscopy was employed to directly compare the chemical structures of pristine waste tea leaves (WTLs) and extracted waste tea leaf microcrystalline cellulose (WTL-MCC), thereby evaluating the effectiveness of the dewaxing, alkali treatment, bleaching, and acid hydrolysis sequence. The comparative spectra are shown in Figure 4. The spectrum of pristine WTLs exhibited characteristic absorption features associated with cellulose, together with contributions from hemicellulose, lignin, and other extractive components. After chemical treatment, substantial changes in the intensity and presence of these characteristic bands were observed in the WTL-MCC spectrum. Both WTLs and WTL-MCC exhibited a broad absorption band in the region of approximately 3330 cm^−1^, corresponding to the O–H stretching vibration of the hydrogen-bonded hydroxyl groups. The O–H band became more prominent in WTL-MCC, which can be associated with the increased exposure of cellulose hydroxyl groups following the removal of the surrounding non-cellulosic constituents. The band near 2900 cm^−1^, corresponding to the C–H stretching vibrations of the CH and CH_2_ groups, was also retained after treatment, confirming the preservation of the cellulose backbone.

The most significant differences between the pristine WTLs and WTL-MCC occurred in the regions associated with hemicellulose and lignin. The absorption band near 1730 cm^−1^, associated mainly with the C=O stretching of acetyl and uronic ester groups in hemicellulose and ester-related groups in lignin, was evident in the untreated WTLs but was substantially reduced in WTL-MCC. This reduction indicates the effective removal of hemicellulose and associated ester-containing components during the alkali and bleaching treatments. Similarly, the aromatic skeletal vibrations associated with lignin in the regions around 1595 and 1510 cm^−1^ were strongly diminished after treatment, providing evidence of substantial delignification. The band near 1240 cm^−1^, associated with the C–O stretching of aryl and acetyl groups originating from lignin and hemicellulose, was also considerably reduced in WTL-MCC compared to pristine WTLs. A weak absorption near 1640 cm^−1^ remained in the treated sample and was attributed primarily to the O–H bending vibration of physically adsorbed moisture.

In contrast, the characteristic cellulose bands became more distinct in WTL-MCC. The absorption near 1430 cm^−1^, associated with symmetric CH_2_ bending and commonly considered a cellulose crystallinity-sensitive band, together with the bands around 1370 and 1315 cm^−1^, corresponding to C–H bending and CH_2_ wagging vibrations, respectively, confirmed the increased prominence of the ordered cellulose structure. The strong bands near 1160 and 1030 cm^−1^, assigned to the C–O–C asymmetric stretching of the β-1,4-glycosidic linkage and pyranose-ring skeletal vibrations, respectively, represent characteristic cellulose fingerprints. The band near 897 cm^−1^, associated with the deformation of the β-glycosidic linkage, was also clearly retained in WTL-MCC and is characteristic of cellulose I. The comparative FTIR spectra demonstrate that the chemical treatment effectively removed substantial portions of hemicellulose and lignin from the pristine WTLs while retaining and enhancing the characteristic spectral features of cellulose. Therefore, the FTIR results provide direct spectroscopic evidence for the conversion of lignocellulosic WTLs into cellulose-rich WTL-MCC reinforcement. These spectral data are consistent with those reported for microcrystalline cellulose extracted from black tea waste by Debnath et al. [49], from Ficus benghalensis leaf by Narayanaperumal et al. [50], and from Lagenaria siceraria pedicles by Asif et al. [51], all of whom reported the loss of the 1730 cm^−1^ carbonyl and aromatic lignin bands together with a strengthened O–H and C–O–C cellulose fingerprint upon removal of hemicellulose and lignin.

### 3.2. X-Ray Diffraction (XRD)

**Figure 5:**
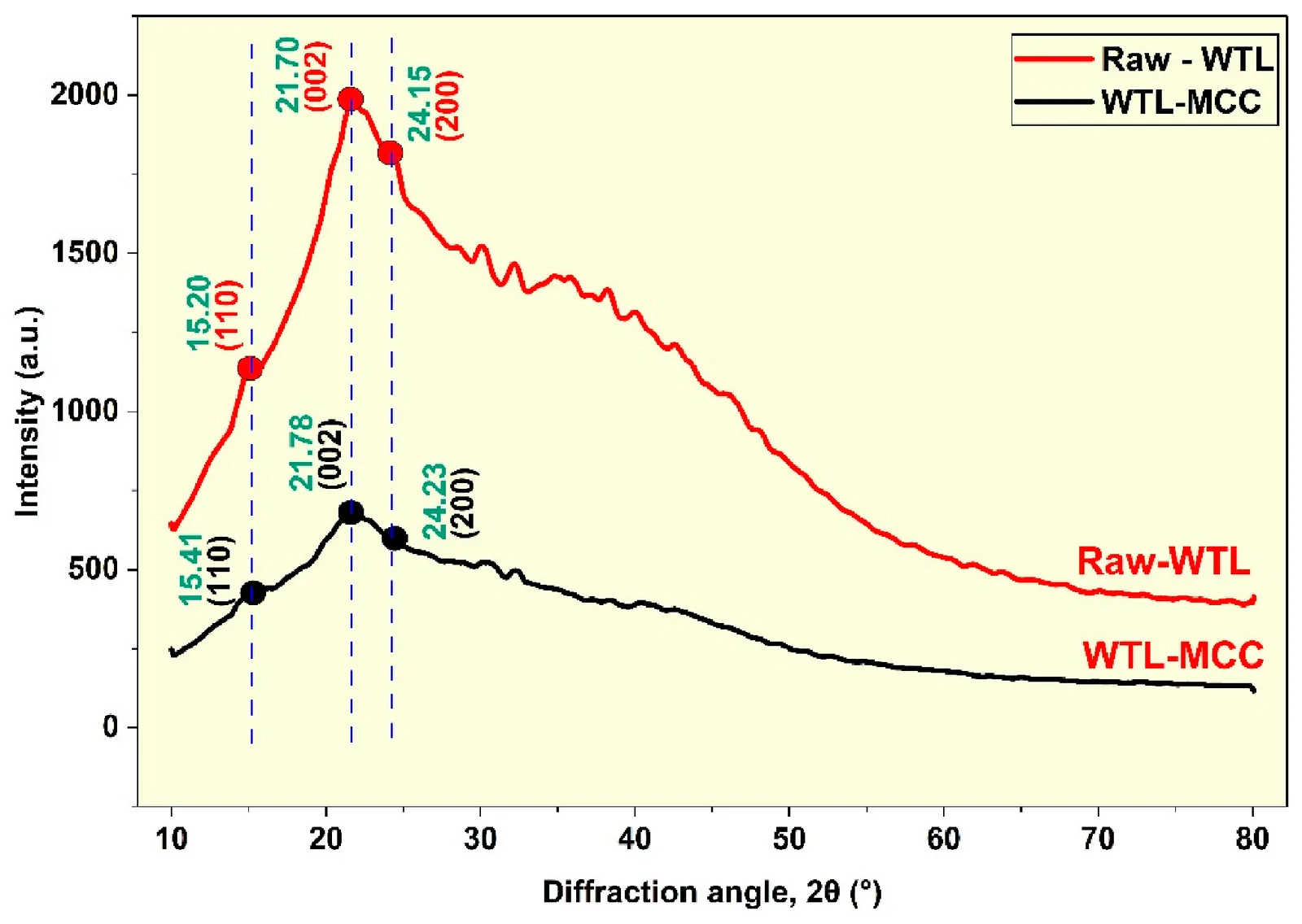
X-ray diffraction (XRD) patterns of pristine waste tea leaves (WTLs) and waste tea leaf microcrystalline cellulose (WTL-MCC).

The X-ray diffraction (XRD) patterns of pristine waste tea leaves (raw WTLs) and waste tea leaf microcrystalline cellulose (WTL-MCC) are presented in Figure 5 to evaluate the structural changes following chemical treatment. The diffractogram of raw WTLs was broad and diffuse, consistent with a heterogeneous lignocellulosic matrix dominated by a crystalline cellulose component, amorphous hemicellulose, lignin and extractives. The diffractogram of WTL-MCC was relatively well defined, indicative of a structurally more ordered cellulose component that was developed following alkali–bleaching–acid hydrolysis. The major diffraction peaks of raw WTLs were observed at 2θ = 15.20°, 21.70°, and 24.15°, whilst those of WTL-MCC were observed at 15.41°, 21.78°, and 24.23°, consistent with the retention of the fundamental cellulose crystalline structure. The peaks in the 15–16° and 21–22° regions are like those observed in MCC derived from black tea waste [49], Lagenaria siceraria fruit pedicles [51] and oil palm empty fruit bunches [52]. The lack of significant 12 cellulose II peaks and the retention of the cellulose I-associated pattern suggest minimal disruption of the native cellulose structure.

The presence of a crystalline (200) reflection on the amorphous background, obtained for WTL-MCC, confirms the semicrystalline morphology of WTL-MCC. Calculated using the Segal method (Equation (1)), the crystallinity index (CrI) for raw WTLs was 31.7%, while the CrI for WTL-MCC increased to 48.1%, equating to an absolute increase of 16.4% and a relative increase of approximately 51.7%. The apparent increase suggests that there is relatively more contribution from the more ordered cellulose domains following removal of the amorphous and non-cellulosic components. Previous reports have found CrI increases of 40.43–64.53% (alkali-treated fiber–MCC [51]) and 70.55% (oil palm, empty-fruit-bunch-derived cellulose [52]) (reported as the intensity ratios I200–I amorphous/I200) because of removal of non-cellulosic material. Tasnim et al. [53] found CrI values of 86.82% and 87.63% for unmodified and standard MCC, respectively, and Debnath et al. [49] found a CrI of 89.77% for black tea waste MCC. Arbelaiz and Orue [54] reported values of 33–51% for NC derived from walnut-shell celluloses, indicating significant variability in the CrI between different biomass sources, as well as variability due to the pretreatment and hydrolysis severity and the CrI determination methods. The modest CrI value of 48.1% for WTL-MCC is in keeping with the much milder (2.5 N HCl, 45 °C, 1 h) hydrolysis used here to preserve the crystalline regions while hydrolyzing the amorphous regions.

The crystallite size of WTL-MCC was calculated to be 2.8 nm based on the FWHM of the main crystalline peak using Scherrer’s formula (Equation (2)). This crystallite size is within the nanometer range, like cellulose materials. According to Susi et al. [52], the crystallite sizes were in the nanometer range, where the size measured as 2.35 nm was under optimal extraction parameters. Also, according to Narayanaperumal et al. [50], the crystallite size of Ficus benghalensis-leaf-derived MCC was 1.56 nm with a crystallinity index of 66.43%. Variations in crystallite size depend on the different biomass origin, treatment parameters, acid concentration, hydrolysis time, and peak broadening.

### 3.3. Thermogravimetric Analysis with Derivative Thermogravimetry (TGA–DTG)

**Figure 6:**
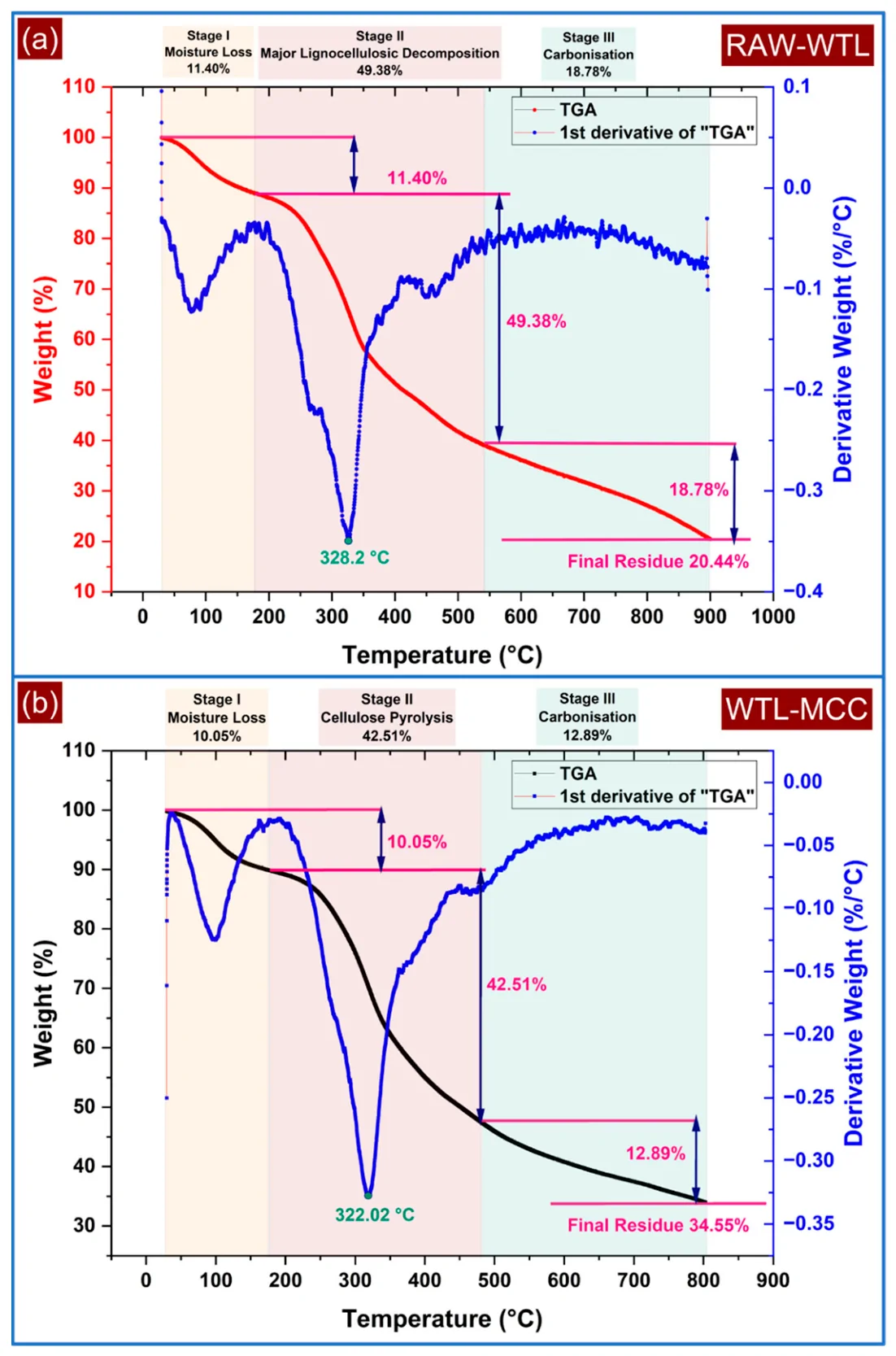
TGA-DTG of (**a**) pristine waste tea leaves (WTLs) and (**b**) waste tea leaf microcrystalline cellulose (WTL-MCC).

The comparative study on the thermally induced decomposition of raw WTLs and WTL-MCC was carried out using TGA-DTG, as illustrated in Figure 6a,b. On the one hand, the first step of decomposition of raw WTLs led to a weight loss of 11.40%, mainly due to the elimination of moisture. This means that the weight reduced from 100% to 88.60%. On the other hand, for WTL-MCC, the first step of decomposition led to a weight loss of 10.05%, reducing from 100% to 89.95%.

This second stage marks the zone where the main thermal degradation takes place. The raw WTLs had a reduction in mass from 88.60% to 39.22%, representing a total mass loss of 49.38% in relation to the initial sample mass. This zone of degradation may be related to the decomposition of heterogeneous components of lignocellulosic nature in raw tea leaves. The corresponding DTG profile exhibited a broad and complex degradation behavior, reflecting the overlapping thermal decomposition of these components. In contrast, WTL-MCC exhibited a lower second-stage mass loss of 42.51%, with the mass decreasing from approximately 90.00% to 47.49%. More importantly, WTL-MCC exhibited a well-defined principal DTG peak at 322.02 °C, whereas raw WTLs showed a broader principal degradation maximum at approximately 328.2 °C. The sharper DTG peak observed for WTL-MCC indicates a more homogeneous, cellulose-rich structure after the removal of non-cellulosic constituents. The appearance of a well-defined cellulose-dominated degradation peak, together with the reduction in the broad overlapping degradation features observed for raw WTLs, provides further evidence that hemicellulose and lignin were effectively removed during the alkali–bleaching–acid hydrolysis sequence, in agreement with the FTIR and XRD results.

The T_max_ of 322.02 °C obtained for WTL-MCC is also comparable with the values reported for agro-waste-derived microcrystalline cellulose. For example, T_max_ values of 319.53 °C for watermelon-rind MCC [55], 296.65 °C for *Rosa indica* petal MCC [56], and 297 °C for banana (*Musa paradisiaca*) leaf MCC [57] have been reported. Therefore, the observed value of 322.02 °C falls within the approximately 300–360 °C range generally reported for the maximum decomposition of purified cellulose, confirming the cellulose-rich nature of the extracted WTL-MCC. The relatively high T_max_ further demonstrates that the extracted material possesses a suitable thermal resistance for subsequent melt processing.

The third stage corresponds to the gradual degradation and carbonization of the thermally stable residue. Raw WTLs underwent an additional 18.78% mass loss, whereas WTL-MCC underwent a mass loss of 12.89%. The final residue was 20.44% for raw WTLs at 900 °C, while WTL-MCC retained 34.55% at 800 °C. The higher residual fraction of WTL-MCC may be associated with the formation and persistence of thermally stable char and inorganic/mineral constituents after the removal of readily degradable organic fractions. Conversely, the lower final residue of raw WTLs, despite being measured at a higher final temperature, indicates continued degradation of residual organic constituents during the high-temperature region.

The comparative TGA–DTG analysis demonstrates a clear transformation in the thermal degradation behavior from the heterogeneous raw WTLs to the more cellulose-dominated WTL-MCC. The principal degradation temperatures were relatively close (328.2 °C for raw WTLs and 322.02 °C for WTL-MCC); however, the distinctly sharper DTG peak of WTL-MCC and its reduced major-stage mass loss indicate increased compositional homogeneity following purification. The reduction in the broad degradation characteristics associated with raw WTLs, together with the FTIR and XRD findings, confirms the effective removal of hemicellulose and lignin during extraction. Furthermore, the Tmax of 322.02 °C is consistent with reported agro-waste MCC values and falls within the typical decomposition range of purified cellulose. The WTL-MCC also retained 34.55% residue at 800 °C, compared with 20.44% for raw WTLs at 900 °C, demonstrating a substantial change in its high-temperature degradation and carbonization behavior. Finally, the thermal stability of WTL-MCC up to approximately 250 °C was adequate for the PLA processing and FDM conditions employed in this study, which were within approximately 180–215 °C.

### 3.4. Differential Scanning Calorimetry (DSC)

**Figure 7:**
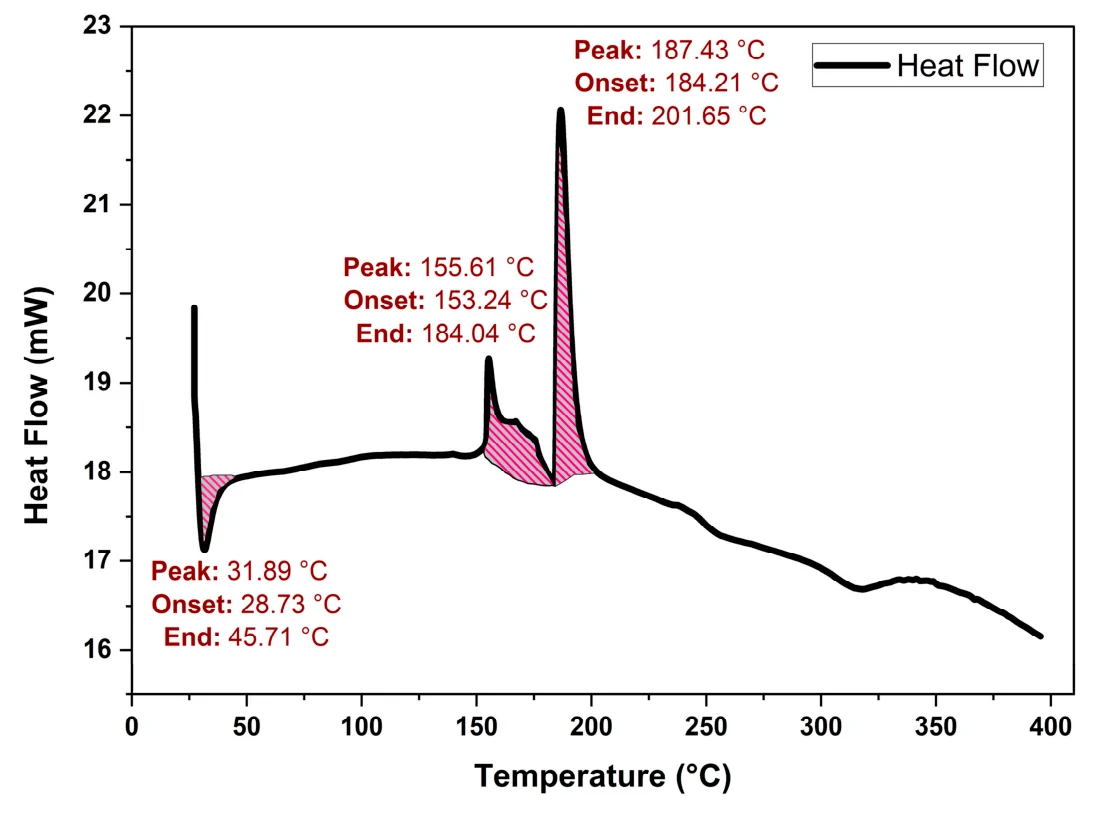
DSC of WTL-MCC.

The DSC thermogram of the extracted WTL-MCC (Figure 7), recorded in the endothermic downward direction, reveals the characteristic thermal behavior of a cellulosic material. An endothermic event was observed at a peak temperature of 31.89 °C (onset 28.73 °C, end 45.71 °C), attributed to the evaporation of loosely bound, physically absorbed water from the hydrophilic cellulose surface; this moisture desorption is an endothermic dehydration process commonly reported for microcrystalline cellulose in the low-temperature region. On further heating, two exothermic peaks were recorded at 155.61 °C (onset 153.24 °C, end 184.04 °C) and 187.43 °C (onset 184.21 °C, end 201.65 °C), the latter being the more intense thermal event. These are not melting transitions because cellulose does not melt but instead undergoes thermal reorganization and degradation; the exothermic nature of the peaks is therefore consistent with solid-state exothermic reactions rather than a first-order melting endotherm. Thermal events in this 150–200 °C region have similarly been reported for MCC by Fouad et al. [58] and were attributed to the amorphous and residual non-cellulosic components of the material.

Accordingly, the exothermic peaks observed here are assigned to the reorganization and early exothermic dehydration/condensation reactions of the less-ordered (amorphous) cellulose fraction, together with a possible contribution from the residual inorganic constituents indicated by the high char residue in the TGA analysis. The absence of an appreciable mass loss in this temperature range in the corresponding TGA curve is consistent with the established observation that DSC thermal events do not need to coincide with the temperatures at which mass loss is detected by TGA. The principal endothermic depolymerization of the cellulose backbone occurred at a considerably higher temperature (300–360 °C), in agreement with the maximum decomposition rate of 322 °C determined from TGA–DTG analysis [59,60], confirming that the WTL-MCC remained thermally stable well above the processing temperatures used for the PLA composites. The DSC results corroborate the FTIR, XRD, and TGA findings, indicating that the extracted material is predominantly crystalline cellulose containing a minor amorphous/residual fraction responsible for the low-temperature exothermic activity.

### 3.5. Ultraviolet–Visible (UV–Vis) Spectroscopy

**Figure 8:**
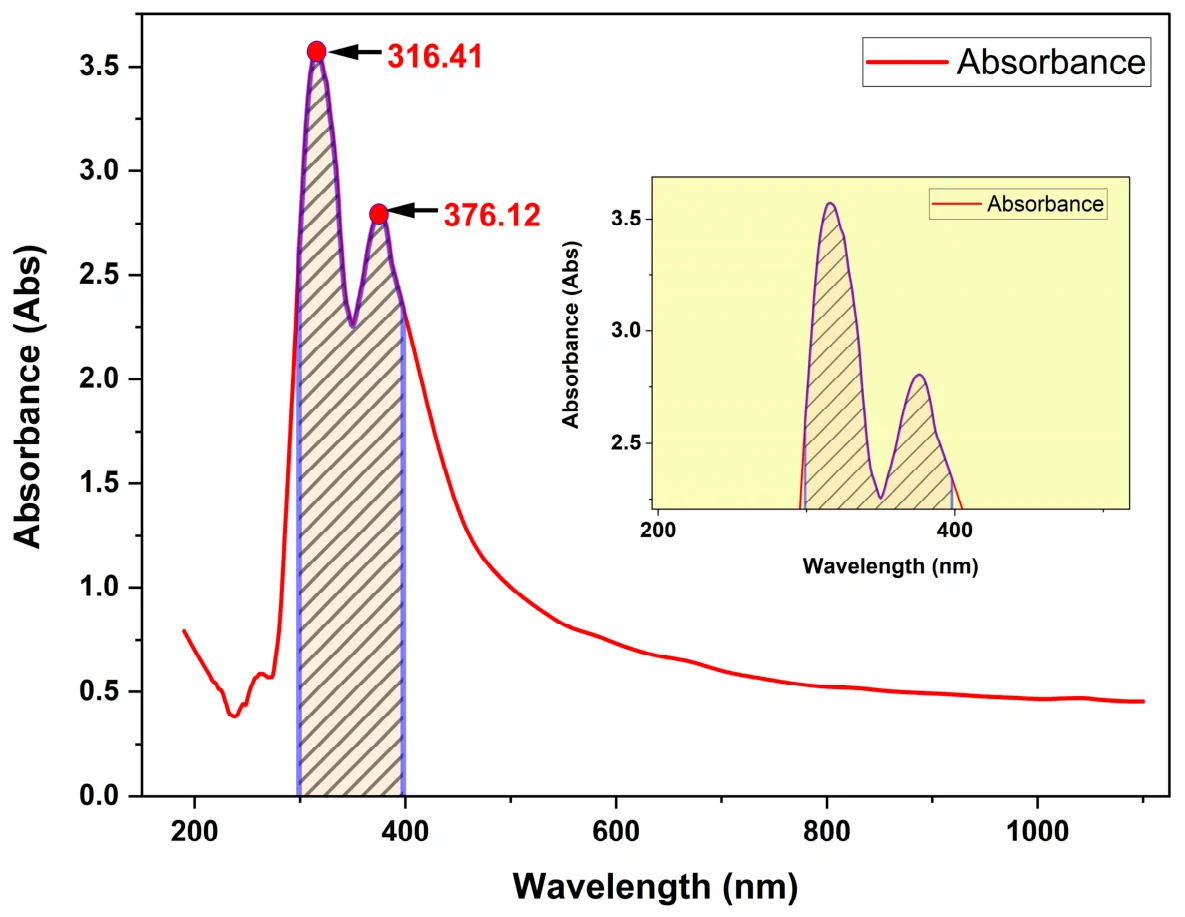
UV analysis of WTL-MCC.

The UV–Vis absorption spectrum of the extracted WTL-MCC (Figure 8) was obtained within the range of 190–1100 nm to study the electronic transitions in the substance and the existence of any leftover chromophoric components. There were two absorption maxima observed within the ultraviolet range at 316.41 nm and 376.12 nm, followed by a gradual decrease in absorption towards the visible range and finally by a featureless baseline extending into the near-infrared region. Since the cellulose structure does not have conjugated or aromatic chromophores, pure cellulose is practically non-absorbent in the ultraviolet and visible region, and the absorption peaks obtained correspond to the π→π* and n→π* electronic transitions of the remaining non-cellulosic chromophoric components.

Specifically, the band at 316.41 nm is attributed to the π→π* transitions of residual aromatic and phenolic structures of lignin origin, which are known to absorb across the 280–350 nm region, while the band at 376.12 nm is ascribed to the n→π* transitions of conjugated carbonyl (C=O) groups and other extended-conjugation species associated with trace phenolic/flavonoid residues. The low and gradually declining absorbance beyond 400 nm, together with the absence of any distinct bands in the visible region, is consistent with the pale color of the WTL-MCC and indicates that the strongly light-absorbing colored impurities of the raw tea leaf were largely removed during the alkali, bleaching, and acid hydrolysis treatments. Nonetheless, the residual absorption in the ultraviolet region revealed that a minor chromophoric fraction persisted, in agreement with the trace lignin band identified by FTIR and the mineral/ash residue observed in the TGA analysis. Comparable ultraviolet absorption behavior has been reported for agro-waste-derived microcrystalline cellulose [61,62], for example, an absorption maximum near 276 nm assigned to a π→π* transition for watermelon-rind MCC [55], whereas extremely pure cellulose has been shown to display almost negligible absorption across this range. Thus, the UV–Vis results confirmed that the extraction sequence produced a predominantly cellulosic material with only a little residual chromophoric content.

### 3.6. Brunauer–Emmett–Teller (BET) Surface Area Analysis of WTL-MCC

**Figure 9:**
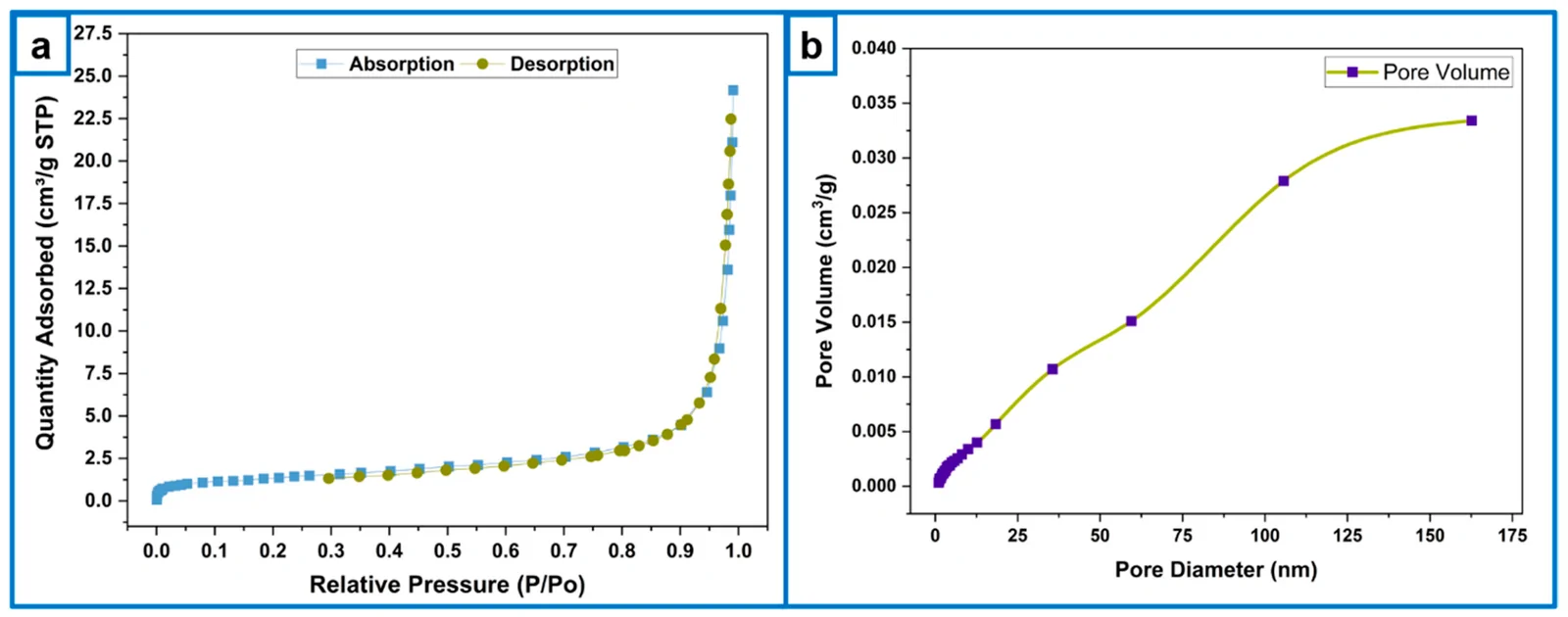
BET analysis of WTL-MCC: (**a**) nitrogen adsorption–desorption isotherm and (**b**) pore size distribution.

The specific surface area and porosity of the extracted WTL-MCC were evaluated from the nitrogen adsorption–desorption isotherm recorded at 77 K (Figure 9a) and the corresponding pore-size distribution (Figure 9b). The isotherm exhibited a low quantity of nitrogen adsorbed at low and intermediate relative pressures, followed by a steep rise as the relative pressure approached saturation (P/P_0_ → 1), accompanied by a narrow hysteresis loop. According to the IUPAC classification, this behavior corresponds to a Type II/Type IV isotherm with an H3-type hysteresis loop, which is characteristic of a predominantly non-microporous solid in which the sharp uptake near saturation arises from capillary condensation within the meso- and macropores formed by the voids between aggregated, plate-like cellulose particles rather than from an intrinsic intraparticle pore network. The narrow H3 loop is consistent with the slit-shaped pores generated by the loose packing of the microcrystalline particles.

The BET specific surface area of WTL-MCC was approximately 5 m^2^/g, and the total pore volume determined from the amount adsorbed near saturation was approximately 0.034 cm^3^/g, with an average pore diameter in the mesopore–macropore range (25–30 nm). Such a low surface area and modest pore volume are typical of microcrystalline cellulose and reflect its dense, highly crystalline structure and aggregation of particles during drying, which limits the accessibility of the internal surface. The pore-size distribution in Figure 9b confirms that the cumulative pore volume increases progressively with the pore diameter and is dominated by larger mesopores and macropores extending up to 160 nm, in agreement with the interparticle origin of the porosity inferred from the isotherm.

While the specific surface area of WTL-MCC may not be high, the existence of pores between the particles enhances its suitability as a reinforcing filler since it ensures effective mechanical bonding and facilitates better wetting and infiltration of molten PLA around the cellulose particles during melt mixing and 3D printing processes. This textural profile is comparable to that of microcrystalline cellulose extracted from other agro-industrial waste materials [63,64] that have relatively low specific surface areas, low bulk density and low porosity between particles.

### 3.7. Scanning Electron Microscopy with Energy-Dispersive X-Ray Analysis (SEM–EDAX)

**Figure 10:**
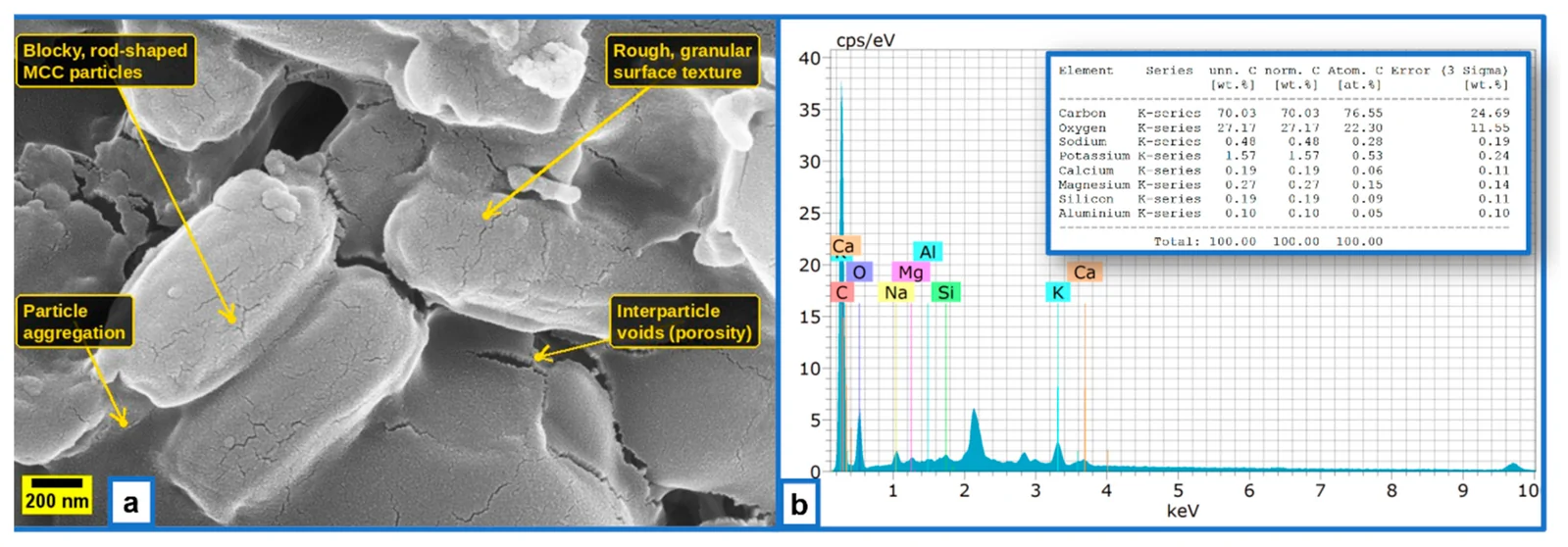
FESEM micrograph and EDAX spectrum of WTL-MCC: (**a**) surface morphology and (**b**) elemental composition.

The morphological characteristics of the isolated WTL-MCC were studied using field-emission scanning electron microscopy. As can be seen in the high-resolution image (Figure 10a), the WTL-MCC comprises a mixture of irregular, blocky, and rod-like microparticles with dimensions at the submicron-to-micron level, which are closely packed and partially aggregated. The particles showed relatively smooth edges but with a granular surface, and there were well-defined interparticle voids among them. Such a particulate morphology is typical for microcrystalline cellulose, which arises due to the elimination of amorphous hemicellulose, lignin, and disordered cellulose molecules through alkali, bleaching, and acid hydrolysis treatments, leading to the disintegration of the fibrous structure of the tea leaf into separate crystallites. The granular surfaces and interparticle porosity of the particles presented in Figure 10a are entirely in line with the low specific surface area and interparticle (mesopore and macropore) character determined from the BET measurements, and both are beneficial for composite preparation due to their effect on mechanical interlocking and facilitation of the infiltration of the PLA matrix into the void spaces.

The results of the elemental analysis obtained using EDAX (Figure 10b) revealed that the material obtained was composed of cellulose. Carbon (70.03 wt.%, 76.55 wt.%) and oxygen (27.17 wt.%, 22.30 wt.%) were the major elements of the obtained material composition, which together made up more than 97% wt. of the total composition due to the carbon–oxygen skeleton of cellulose; the higher percentage of carbon is due to the conductive carbon coating of the specimen. Importantly, no sulfur or chlorine was detected, indicating that the sodium chlorite bleaching and hydrochloric acid hydrolysis residues were effectively removed during the repeated washing steps. Nevertheless, a small proportion of inorganic elements was present, namely potassium (1.57 wt.%), sodium (0.48 wt.%), magnesium (0.27 wt.%), calcium (0.19 wt.%), silicon (0.19 wt.%), and aluminum (0.10 wt.%). The comparatively high potassium, together with calcium and magnesium, originates from the minerals naturally accumulated in the tea leaf, whereas sodium is a residue of the alkali treatment. This residual inorganic (ash) fraction of approximately 2.8 wt.% is fully consistent with, and directly accounts for, the high char residue of 34.55% observed in the TGA analysis and the residual ultraviolet absorption noted in the UV–Vis spectrum. Comparable SEM–EDAX results, showing rod-like or blocky microcrystalline particles composed predominantly of carbon and oxygen with minor mineral residues, have been reported for microcrystalline cellulose extracted from black tea waste [49], Ficus benghalensis leaves [50], watermelon rinds [55], and Rosa indica petals [56].

### 3.8. Atomic Force Microscopy (AFM) of WTL-MCC

**Figure 11:**
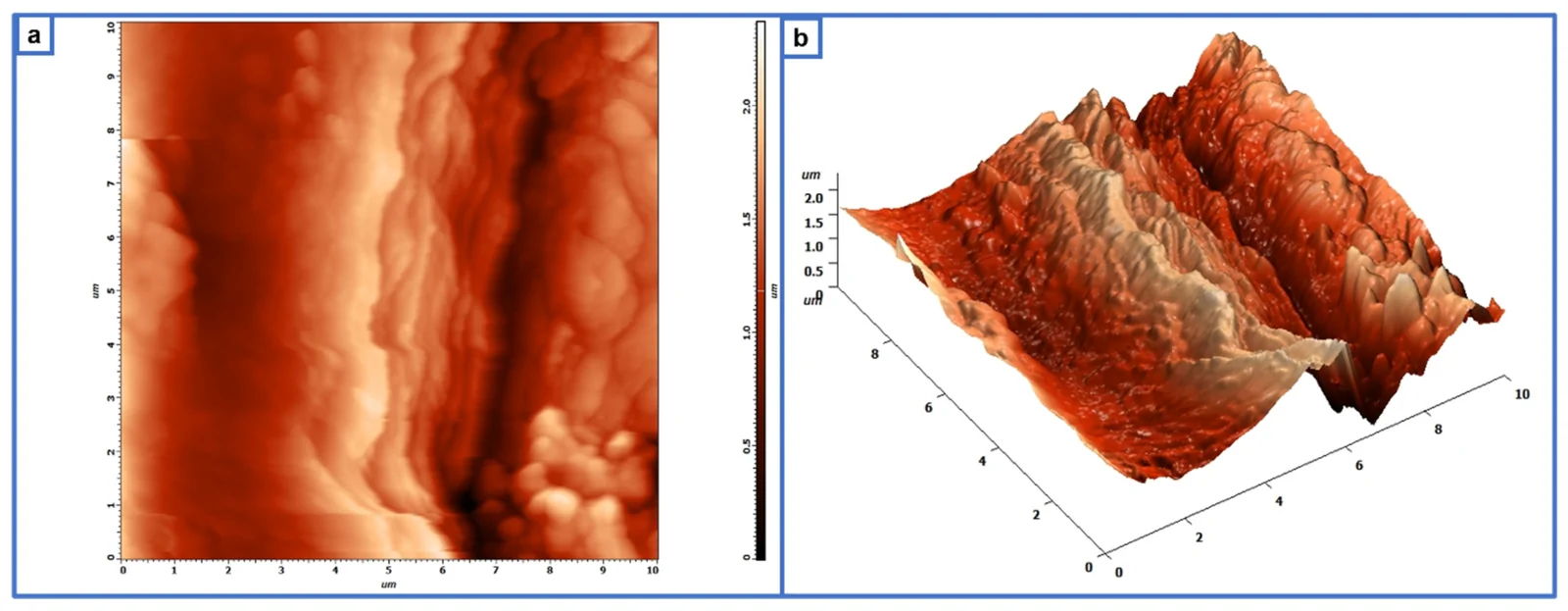
AFM surface topography of WTL-MCC: (**a**) two-dimensional (2D) height image and (**b**) three-dimensional (3D) surface morphology.

The surface topography and nanoscale roughness of the extracted WTL-MCC were characterized by atomic force microscopy over a 10 × 10 µm scan area, and the two- and three-dimensional height images are presented in Figure 11a,b. The images reveal an undulating, granular surface consisting of a succession of peaks and valleys that correspond to the aggregated microcrystalline particles observed by SEM, with the maximum height variation (peak-to-peak, S_y_) reaching 2365.75 nm across the scanned region. The average roughness (S_a_) and root-mean-square roughness (S_q_) were determined to be 285.996 nm and 344.194 nm, respectively, while the ten-point height (S_z_) and the mean height were 1160.86 nm and 1285.99 nm. These relatively high roughness values reflect the particulate aggregated morphology of microcrystalline cellulose, in which micron-scale crystalline particles produce large excursions over the scanned area.

Roughness statistical characteristics offer additional information about the nature of the surface. The value of surface skewness (S_sk_) was negative (−0.169); thus, the height distribution had slightly more valleys and depressions below the mean plane compared to peaks above the mean plane. Skewness is an inherent property of a porous surface morphology and was found in microcrystalline cellulose from other agro-waste, which was also related to the porosity of the surface. The excess kurtosis (S_ku_) value was negative as well (−0.433), and this implies a platykurtic height distribution. A low kurtosis value is typical for a bumpy surface, while a high kurtosis value indicates a spiky surface. This proves that the WTL-MCC surface comprises bumpy elements rather than spikes, as shown in the SEM images of the smooth outline of the particles. High surface roughness, valley-dominant (porous) skewness, and bumpy morphology are consistent with the interparticle porosity revealed by the BET method and rough granular particle surfaces in SEM images.

From an application perspective, this rough and porous surface topography is highly favorable for the use of WTL-MCC as a reinforcing filler. The increased surface area and roughness enhanced the physical contact and mechanical interlocking between the cellulose particles and the PLA matrix, promoting stronger interfacial adhesion and more efficient stress transfer within the 3D-printed composite. Comparable AFM roughness behavior, including negative skewness and roughness parameters supporting good interfacial bonding, has been reported for microcrystalline cellulose extracted from *Eucalyptus tereticornis* leaves [63], watermelon rind [55], and vegetable-waste biomass [65].

### 3.9. Tensile Properties of the WTL-MCC/PLA Composites

**Figure 12:**
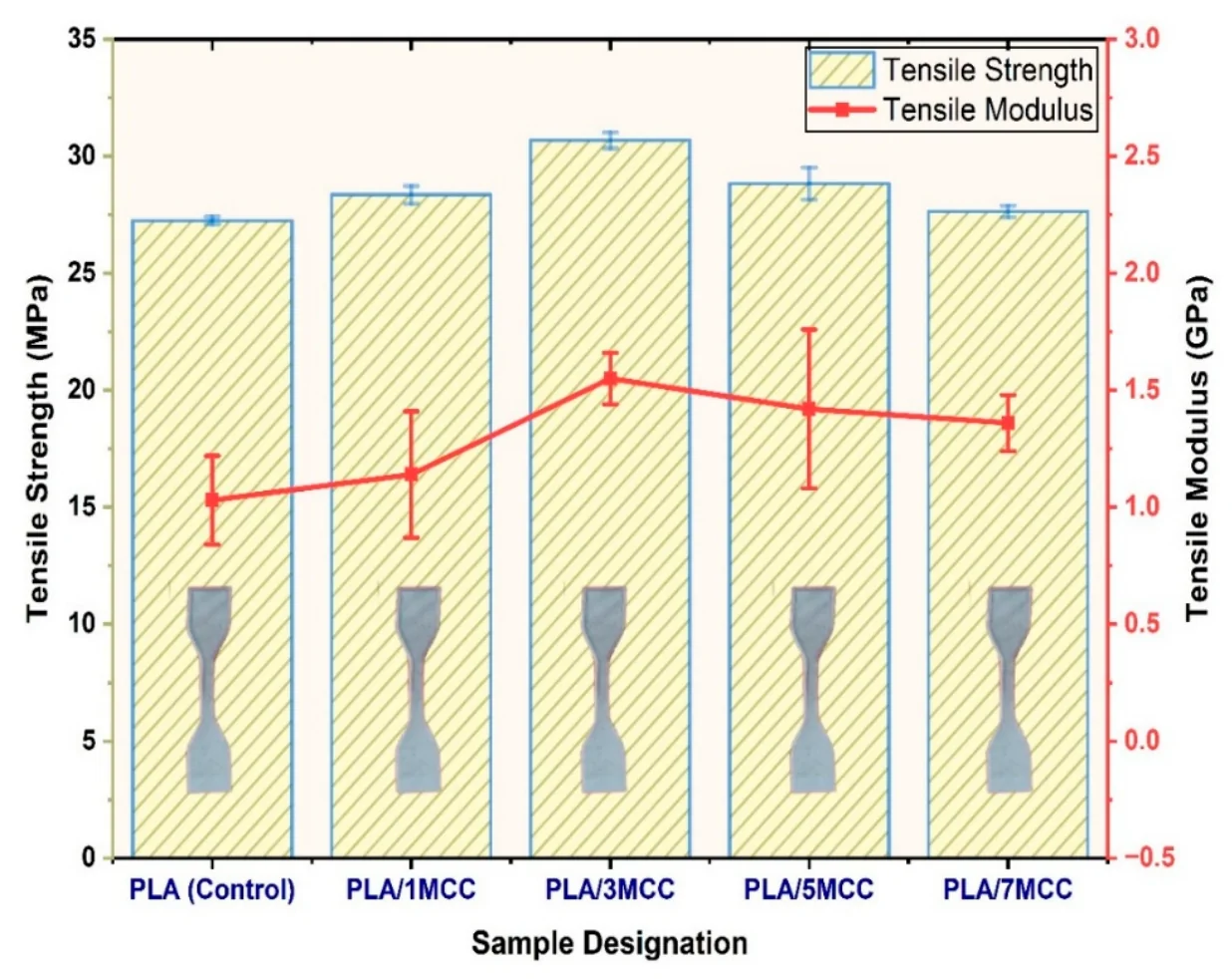
Tensile properties of the WTL-MCC/PLA composites.

The tensile strength and tensile modulus of the 3D-printed neat PLA and WTL-MCC/PLA composites were evaluated according to ASTM D638, and the results (mean of the three specimens) are shown in Figure 12. Both properties exhibited a clear dependence on the WTL-MCC loading, increasing to a maximum at 3 wt.% and then decreasing at higher loadings while remaining at or above the values of neat PLA. The neat PLA recorded a tensile strength of 27.25 MPa and a tensile modulus of 1.03 GPa. With the incorporation of WTL-MCC, the tensile strength rose progressively to a peak of 30.68 MPa at 3 wt.% (an increase of 12.6% relative to neat PLA) and thereafter declined to 28.84 MPa at 5 wt.% and 27.64 MPa at 7 wt.%. The tensile modulus followed the same trend, reaching a maximum of 1.55 GPa at 3 wt.% (an increase of 50.5%) before decreasing to 1.42 GPa and 1.36 GPa at 5 and 7 wt.%, respectively; notably, the modulus of all the composites remained substantially higher than that of the neat PLA even at the highest loading.

Improvements in both tensile strength and modulus up to 3 wt.% are attributed to the effective reinforcing action of the well-dispersed WTL-MCC particles. At low loadings, the rigid, highly crystalline cellulose particles are uniformly distributed within the PLA matrix and aided by hydrogen bonding between the abundant surface hydroxyl groups of the cellulose (confirmed by FTIR) and the carbonyl groups of PLA; together with the mechanical interlocking promoted by the rough, porous particle surface (observed by SEM and AFM), they establish a strong filler–matrix interface. This enables efficient stress transfer from the compliant matrix to the stiff crystalline filler and restricts the mobility of the polymer chains, thereby increasing both the strength and stiffness. The reinforcing effect is further reinforced by the nucleating action of the WTL-MCC on the crystallization of the PLA phase, as revealed by the DSC results.

When the loading exceeded an optimum value of 3 wt.%, the tensile strength dropped and came close to that of pure PLA. Such behavior is typical of particulate biocomposites and is caused by the agglomeration of cellulose particles at high contents due to the strong hydrogen bonding between the hydrophilic cellulose particles [21]. The agglomerates serve as stress concentrators and create areas of poor interfacial adhesion. It is significant, however, that the tensile modulus remained well above that of neat PLA at all loadings; because stiffness is governed primarily by the volume fraction and intrinsic rigidity of the crystalline filler rather than by the quality of the interface, the incorporation of stiff WTL-MCC continued to increase the composite modulus even when agglomeration limited the strength. Comparable decoupling, in which cellulosic fillers increase the modulus while the tensile strength decreases at higher loadings, has been widely reported for 3D-printed cellulose–PLA systems [66].

The tensile behavior observed here is fully consistent with the recent literature on additively manufactured cellulose/PLA composites. In particular, the optimum microcrystalline cellulose content of 3 wt.% giving the best tensile strength and modulus, with reduced performance and heterogeneous dispersion at higher loadings, has been reported for 3D-printed PLA/MCC composites, while similar optimum-loading behavior has been observed at 2.5 wt.% for PLA/cellulose-nanofibril filaments and at low loadings for MCC-modified wood–PLA filaments. The tendency of cellulosic fillers to increase the modulus even as the tensile strength decreases at higher loadings, owing to agglomeration and void formation, has likewise been documented for 3D-printed PLA reinforced with microcrystalline cellulose, cellulose nanofibrils, and natural fibers. Therefore, the present WTL-MCC/PLA composites display a well-defined optimum reinforcement at 3 wt.%, confirming that the WTL-MCC is an effective bio-based reinforcement for fused deposition modeling (FDM) of PLA.

### 3.10. Flexural Properties of the WTL-MCC/PLA Composites

**Figure 13:**
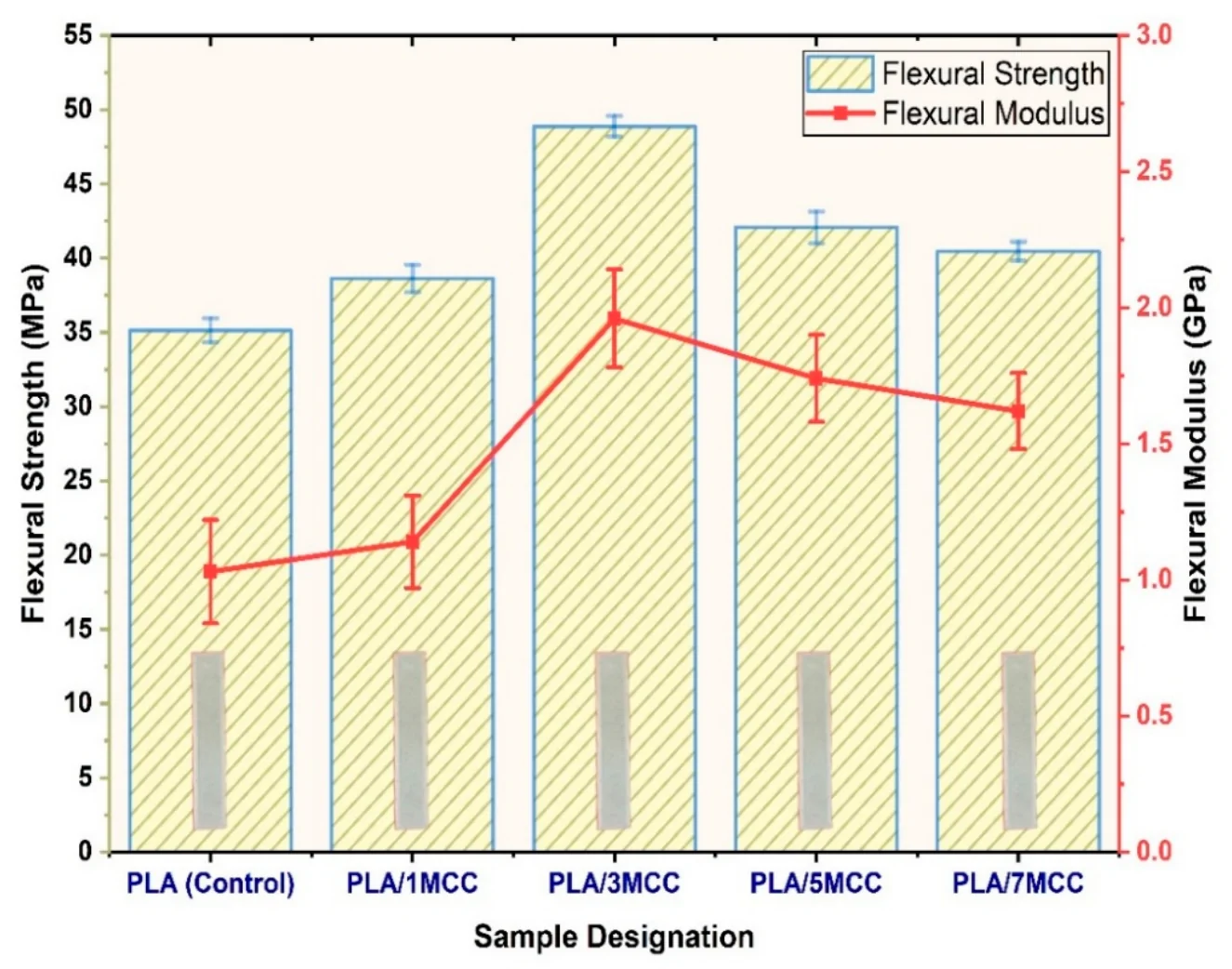
Flexural properties of the WTL-MCC/PLA composites.

The flexural behavior of the 3D-printed neat PLA and WTL-MCC/PLA composites was assessed by three-point bending according to ASTM D790, and the mean flexural strength and flexural modulus (three specimens each) are presented in Figure 13. As with the tensile properties, both the flexural strength and flexural modulus increased markedly with the addition of WTL-MCC, reaching a maximum at 3 wt.%, and then decreased at higher loadings while remaining well above the values of the neat PLA. The neat PLA exhibited a flexural strength of 35.14 MPa and a flexural modulus of 1.03 GPa. Incorporation of WTL-MCC increased the flexural strength to a maximum of 48.87 MPa at 3 wt.%, corresponding to a substantial improvement of 39.1% over the neat PLA, after which it decreased to 42.07 MPa (+19.7%) at 5 wt.% and 40.46 MPa (+15.1%) at 7 wt.%. The flexural modulus displayed the same optimum, rising sharply to 1.96 GPa at 3 wt.%, an increase of 90.3% relative to the neat PLA, before decreasing to 1.74 GPa and 1.62 GPa at 5 and 7 wt.%, respectively, yet still remaining 57% above the neat PLA at the highest loading.

A pronounced enhancement in the flexural properties up to 3 wt.% reflects the same reinforcing mechanisms responsible for the tensile behavior. The uniformly dispersed, rigid crystalline WTL-MCC particles formed a strong interface with the PLA matrix through hydrogen bonding between the cellulose hydroxyl groups and the PLA carbonyl groups and through mechanical interlocking at the rough, porous particle surfaces, as revealed by SEM and AFM. Under bending, the applied load is concentrated in the outer surface layers of the specimen, where efficient stress transfer to stiff cellulose crystallites most effectively resists deformation and crack propagation. Consequently, the relative gains in flexural strength and modulus (+39.1% and +90.3%) are even larger than those recorded in tension (+12.6% and +50.5%), a difference commonly observed for particulate cellulose reinforcements, because bending is more sensitive to stiffening of the surface region. The nucleating action of WTL-MCC, which increases the crystallinity of the PLA matrix (DSC), further contributes to the higher flexural stiffness.

The reduction in the flexural strength and modulus above 3 wt.% is again ascribed to the agglomeration of the hydrophilic cellulose particles at higher loadings, which generates stress-concentrating clusters, interfacial voids, and regions of incomplete matrix wetting that promote earlier crack initiation under bending. As with the tensile modulus, however, the flexural modulus remained substantially above that of the neat PLA at all compositions because the intrinsic rigidity and volume fraction of the crystalline filler continue to stiffen the composite even when the interfacial quality deteriorates. This optimum-loading flexural response, with a maximum at low cellulose content followed by a decline attributed to agglomeration, is in good agreement with recent reports on 3D-printed microcrystalline cellulose/PLA and natural fiber/PLA composites, in which the best flexural performance is likewise achieved at low filler contents, and modulus enhancements are retained even when the strength diminishes [67]. The present results confirm that 3 wt.% is the optimum WTL-MCC loading for maximizing both the tensile and flexural performance of the fused-filament-fabricated composites.

### 3.11. Compressive Properties of the WTL-MCC/PLA Composites

**Figure 14:**
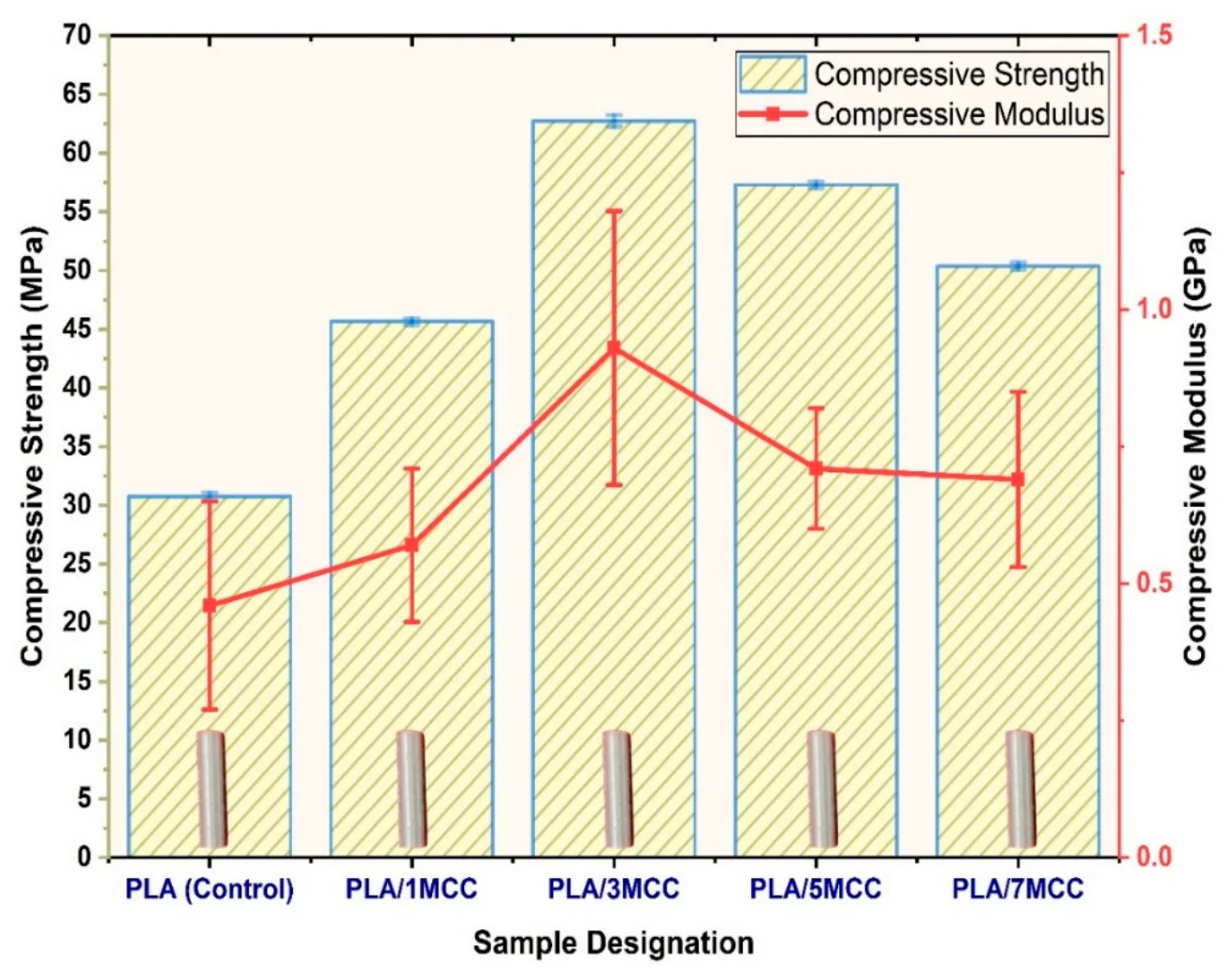
Compressive properties of the WTL-MCC/PLA composites.

The compressive strength and compressive modulus of the 3D-printed neat PLA and WTL-MCC/PLA composites were determined according to ASTM D695, and the mean values of the three specimens are shown in Figure 14. The compressive properties exhibited the same optimum loading behavior as observed in the tensile and flexural tests, increasing to a maximum at 3 wt.% WTL-MCC and then decreasing at higher loadings, while remaining far above that of neat PLA. The neat PLA recorded a compressive strength of 30.76 MPa and a compressive modulus of 0.46 GPa. With the addition of WTL-MCC, the compressive strength increased sharply to 45.65 MPa at 1 wt.% (+48.4%) and reached a maximum of 62.74 MPa at 3 wt.%, corresponding to a remarkable improvement of 104.0%, more than double that of the neat PLA. Beyond this optimum, the compressive strength decreased to 57.29 MPa (+86.2%) at 5 wt.% and 50.36 MPa (+63.7%) at 7 wt.%. The compressive modulus exhibited a similar trend, increasing to reach a peak value of 0.93 GPa at 3 wt.% (+102.2%) and then decreasing to values of 0.71 GPa and 0.69 GPa at 5 wt.% and 7 wt.%, respectively, while still being about 50% higher than neat PLA.

The exceptional enhancement in the compressive properties up to 3 wt.% was a direct consequence of the reinforcing action of the rigid, highly crystalline WTL-MCC particles and their strong interfacial coupling with the PLA matrix. Under compressive loading, the applied stress is distributed throughout the bulk of the specimen and is borne largely by the stiff cellulose crystallites, which resist densification and collapse of the printed raster structure. The uniformly dispersed particles, bonded to the matrix through hydrogen bonding between the cellulose hydroxyl groups and the PLA carbonyl groups (FTIR) and interlocked at their rough, porous surfaces (SEM and AFM), restrict the local deformation and buckling of the polymer chains and effectively transfer the compressive load, thereby producing large gains in both compressive strength and modulus. The particularly high improvement in compressive strength, greater than that recorded in tension or flexure, reflects the fact that compressive failure in fused-filament-fabricated parts is governed by the resistance of the material to inter-layer and intra-raster collapse, which is strongly reinforced by the incorporation of a rigid cellulose filler. The reduced void content and improved packing afforded by the well-dispersed particles at the optimum loading further enhanced this resistance.

The decrease in the compressive properties above 3 wt.% is again attributed to the agglomeration of the hydrophilic cellulose particles, which introduces stress-concentrating clusters, interfacial voids, and poorly wetted regions that act as initiation sites for localized crushing and buckling under compression. As observed for the tensile and flexural moduli, the compressive modulus nevertheless remained well above that of the neat PLA at all compositions because the intrinsic rigidity and volume fraction of the crystalline filler continue to stiffen the composite even when the interfacial quality is diminished by agglomeration. This optimum-loading compressive response is consistent with recent studies on additively manufactured microcrystalline cellulose/PLA composites, in which the incorporation of MCC markedly increased the compressive strength of the printed parts, and with the general behavior of natural filler/PLA composites, where the best mechanical performance is achieved at a low, well-dispersed filler content. Therefore, the compressive results reinforce the conclusion that 3 wt.% is the optimum WTL-MCC loading, at which the tea-leaf-derived microcrystalline cellulose delivers its maximum reinforcing effect across all three modes of mechanical loading.

### 3.12. Acoustic Properties (Sound Absorption Coefficient and NRC)

**Figure 15:**
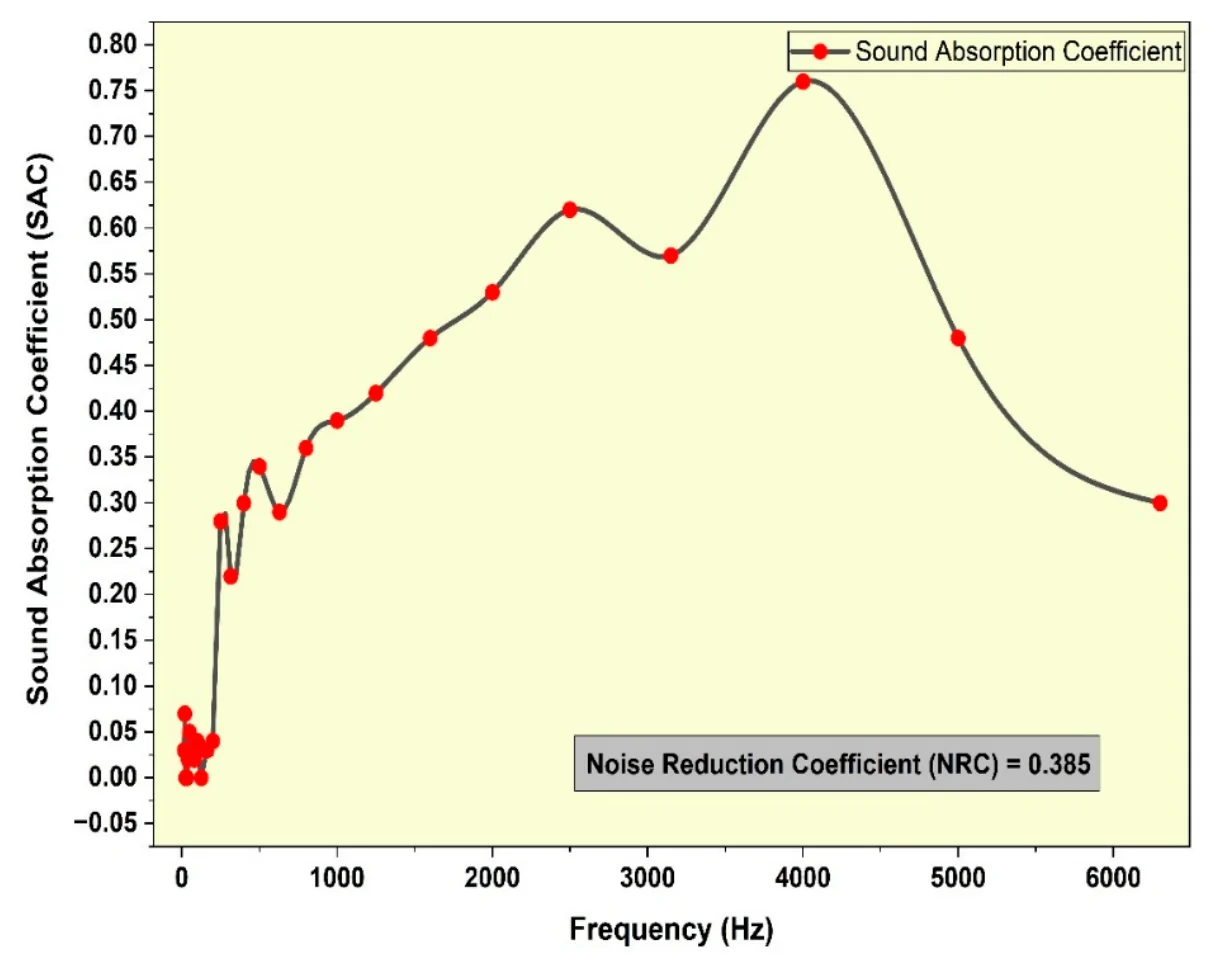
Sound absorption properties of the PLA/3MCC composite.

Because the PLA/3MCC composite exhibited the best overall mechanical performance among all the formulations, recording the highest tensile, flexural, and compressive strengths and moduli, and thereby identifying 3 wt.% as the optimum WTL-MCC loading, this optimized composition was selected as the representative sample for the acoustic evaluation. The sound absorption behavior of the PLA/3MCC composite was determined by the impedance-tube method (ASTM E1050) over the frequency range of 16–6300 Hz, and the resulting sound absorption coefficient (SAC, α) spectrum is presented in Figure 15. At low frequencies (below 200 Hz), the absorption coefficient was very low (α < 0.07), which is typical of rigid, dense panels whose high stiffness prevents the vibrational response required to dissipate low-frequency acoustic energy. Above 250 Hz, the absorption increased sharply and then rose steadily with frequency, reaching 0.39 at 1000 Hz and 0.53 at 2000 Hz before attaining a first maximum of 0.62 at 2500 Hz. Following a slight dip near 3150 Hz (α = 0.57), the absorption increased to its highest value of 0.76 at 4000 Hz and thereafter decreased at higher frequencies. The composite therefore displays a predominantly mid-to-high-frequency absorption character, with the absorption coefficient exceeding 0.5 across the acoustically important 2000–4000 Hz band, which coincides with the sensitivity range of human hearing and the dominant components of speech and environmental noise.

For the composite of PLA and 3MCC, the noise reduction coefficient (NRC), which is the arithmetic average of the absorption coefficients at 250, 500, 1000 and 2000 Hz, was found to be 0.385. This value reflects the moderate broadband absorption of the material and is a realistic result for a solid, three-dimensionally printed structural biocomposite with a high infill density rather than a dedicated porous acoustic absorber. The absorption achieved originates from the internal architecture of the printed composite and the incorporated cellulose: the interfaces and micro-voids between the printed rasters and layers, together with the interparticle porosity and rough surfaces contributed by the WTL-MCC (evidenced by the BET, SEM, and AFM analyses), promote the scattering and viscous dissipation of the incident sound waves, while the rigid cellulose crystallites introduce impedance mismatches and additional internal friction that convert part of the acoustic energy into heat. These mechanisms become progressively more effective as the acoustic wavelength approaches the scale of the internal features, which accounts for the increase in absorption at mid and high frequencies.

Although the overall NRC of 0.385 is below the value of 0.5, generally associated with dedicated commercial sound-absorbing materials, it is a favorable outcome for a dense, load-bearing 3D-printed component, indicating that the optimized WTL-MCC/PLA composite combines useful acoustic damping with its primary structural function. The strong absorption obtained in the 2000–4000 Hz region suggests that the material has practical potential for mid- to high-frequency noise attenuation in multifunctional and semi-structural applications. Comparable moderate NRC values and mid-to-high-frequency-dominated absorption have been reported for other rigid natural-fiber- and cellulose-reinforced polymer composites, confirming that the incorporation of bio-based fillers, such as WTL-MCC, can impart a meaningful acoustic-damping capability to otherwise acoustically reflective thermoplastics.

### 3.13. Fractographic and Elemental Analysis of the WTL-MCC/PLA Composites (SEM–EDS)

**Figure 16:**
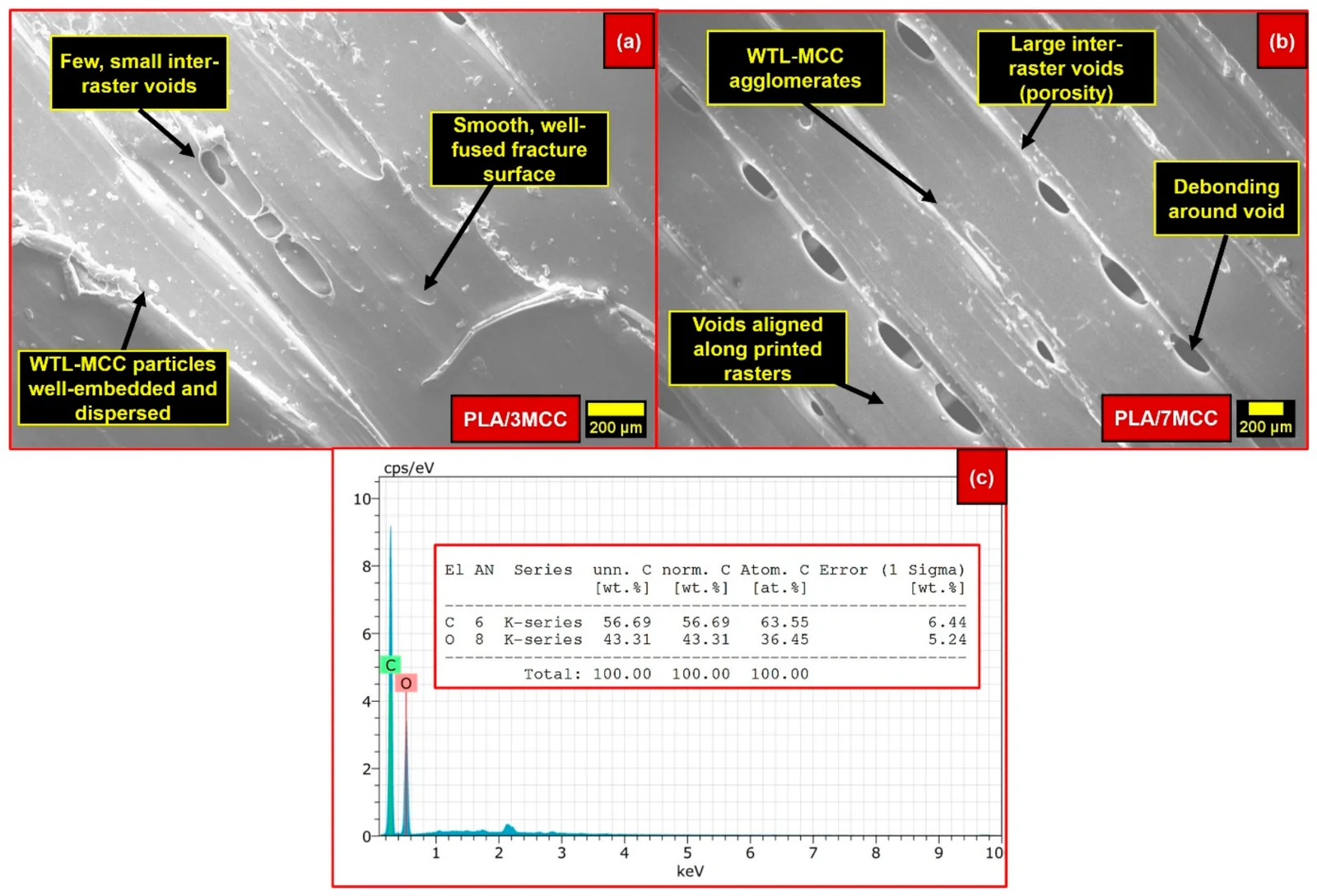
Fractographs of WTL-MCC/PLA composites (**a**) PLA/3MCC and (**b**) PLA/7MCC and (**c**) elemental analysis of PLA/3MCC.

To relate the mechanical behavior of the composites to their underlying microstructure, the tensile fracture surfaces of the best-performing (PLA/3MCC) and poorest-performing (PLA/7MCC) compositions were examined by field-emission scanning electron microscopy, and the elemental composition of the optimum PLA/3MCC composite was determined by energy-dispersive X-ray spectroscopy (EDS). The annotated micrographs are presented in Figure 16a,b, and the EDS data for PLA/3MCC are presented in Figure 16c. The fracture surface of the PLA/3MCC composite (Figure 16a) was smooth, continuous, and well consolidated, showing good fusion between the deposited beads with only a few small, isolated inter-raster voids. The WTL-MCC appeared as fine, bright particles that were uniformly dispersed and firmly embedded within the PLA matrix, with no discernible gaps or pull-out cavities at the particle–matrix boundary, indicating strong interfacial adhesion. The directional flow lines and localized matrix tearing across the surface are characteristic of a tortuous crack path and reflect a higher fracture energy and efficient stress transfer from the ductile matrix to the rigid, well-bonded cellulose crystallites. This well-integrated microstructure was fully consistent with the maximum tensile, flexural, and compressive properties measured for the 3 wt.% composite.

In contrast, the fracture surface of the PLA/7MCC composite (Figure 16b) was dominated by numerous large, elongated voids arranged in regular rows along the printed raster direction, corresponding to the inter-bead gaps that formed when the higher cellulose content increased the melt viscosity and impaired the coalescence and fusion of the deposited filaments. The agglomerations of the excess WTL-MCC appeared as bright clusters near these pores, with evidence of interfacial debonding and cavitation around the agglomerated particles. Agglomerations behave as stress concentrators, while the larger inter-raster voids create a continuous path of pre-existing cracks leading to early failure of the material when subjected to applied loads. It is this poor bead fusion, agglomeration, and high porosity that explain the drop in strength and brittleness seen in the 7 wt.% composite material, indicating that agglomeration and void formation are indeed the predominant modes of failure above the optimal loading.

The result of the EDS analysis for the PLA/3MCC composite (Figure 16c) showed that the fracture surface consisted only of carbon (56.69 wt.%, 63.55 wt.%) and oxygen (43.31 wt.%, 36.45 wt.%), which are the elements forming the organic structure of both the PLA and cellulose. The corresponding carbon-to-oxygen atomic ratio of approximately 1.7 is consistent with the PLA-dominated matrix, the slight carbon enrichment being attributable to the conductive coating applied for imaging. Notably, no inorganic or mineral elements were detected, in contrast to the trace potassium, sodium, calcium, magnesium, silicon, and aluminum identified in the neat WTL-MCC by EDAX. Their absence in the composite is a consequence of both low cellulose loading (3 wt.%), which dilutes these residual minerals below the detection limit, and the homogeneous dispersion of the filler, which confirms that the composite fracture surface is free of contamination or mineral segregation. Taken together, the SEM–EDS results corroborate the mechanical findings: the uniform dispersion; strong interfacial bonding; and dense, well-fused microstructure achieved at the optimum 3 wt.% loading were replaced at 7 wt.% by agglomeration, interfacial debonding, and extensive inter-raster porosity, which govern the deterioration of the mechanical performance.

## 4. Conclusions and Future Scope

In this study, industrial waste tea leaves were successfully valorized into microcrystalline cellulose and used as a renewable reinforcement for 3D-printable PLA composites, demonstrating a complete waste-to-product workflow. The alkali–bleaching–acid hydrolysis route yielded a cellulose I material of moderate crystallinity (CrI = 48%, crystallite size = 2.8 nm) that was thermally stable up to 250 °C, above the FDM processing window, with only a minor residual mineral fraction. The WTL-MCC was uniformly incorporated into PLA and extruded into dimensionally consistent filaments (1.75 ± 0.05 mm, >97% yield) that printed reliably at all loadings. The mechanical performance peaked at 3 wt.% WTL-MCC, where the tensile, flexural and compressive strengths increased by 12.6%, 39.1% and 104.0% and the respective moduli by 50.5%, 90.3% and 102.2% relative to neat PLA, driven by good filler dispersion, strong hydroxyl-mediated interfacial bonding and the nucleating action of the cellulose. Beyond 3 wt.%, agglomeration, increased melt viscosity and aligned inter-raster voids reduced the strength, confirmed by SEM fractography, although the moduli remained above neat PLA at every loading. The optimized PLA/3MCC composite also provided useful mid-to-high-frequency sound absorption (αmax 0.76 at 4000 Hz; NRC 0.385), making it a genuinely multifunctional material.

Waste-tea-derived MCC is confirmed as an effective, low-cost and sustainable reinforcement for FDM-printed PLA, with 3 wt.% giving the best balance of dispersion, bonding, stiffness and acoustic damping. These attributes suit lightweight semi-structural and multifunctional applications, including consumer and electronic housings, automotive and interior trim panels, acoustic and vibration-damping components, and customized architectural elements where load-bearing capacity, noise attenuation, and biodegradability are jointly desirable. Future work should focus on surface functionalization or compatibilization of WTL-MCC to suppress agglomeration and extend the reinforcing window beyond 3 wt.%, fuller removal of residual minerals, and reduction in inter-raster porosity through optimized print parameters. Broader mechanical testing (impact, fatigue, dynamic mechanical analysis), acoustic optimization using porous or lattice infills to raise the NRC, and life cycle and biodegradability assessments are also recommended.

### Limitations and Future Work

Several limitations are inherent to this study, which, in turn, point out certain directions for future research. First, it should be noted that the purity of the WTL-MCC was assumed based on the FTIR, XRD, TGA and EDAX results; however, the purity could be confirmed through the quantification of the amounts of cellulose, hemicellulose and lignin in the sample with the help of the standard compositional analysis (TAPPI/Van Soest method). The next limitation lies in the fact that the porosity reduction and improved packing at the optimal loading were considered qualitatively by examining the fracture surface. It would be reasonable to measure the porosity and density of the material quantitatively by means of, for example, image analysis, micro-CT or gas/geometric pycnometry. Moreover, no melt viscosity measurements have been carried out to confirm the explanation provided for the worse inter-raster bonding at high loadings. Thus, the melt flow rate measurement and/or rheological investigation of the composite could be suggested. Lastly, a complete stress–strain curve and ANOVA statistical analysis of the mechanical data, in addition to the acoustic optimization, should be mentioned.

## Figures and Tables

**Table 1:** Composition of neat PLA and WTL-MCC/PLA composite filaments.

Sample Designation	PLA (wt.%)	WTL-MCC (wt.%)
PLA (Control)	100	0
PLA/1MCC	99	1
PLA/3MCC	97	3
PLA/5MCC	95	5
PLA/7MCC	93	7

**Table 2:** FDM printing parameters for WTL-MCC/PLA composite test specimens.

Parameter	Value
Printer	Ender-3 (Creality, China)
Slicing software	Ultimaker Cura (v5.1.0)
Nozzle diameter	0.4 mm
Nozzle temperature	215 °C
Build plate temperature	60 °C
Layer thickness	0.20 mm
Infill pattern	Rectilinear
Infill density	100%
Print speed	30 mm s^−1^
Travel speed	60 mm s^−1^

## Data Availability

The original contributions presented in this study are included in the article. Further inquiries can be directed to the corresponding author.

## Symbol/Abbreviation

Symbol/Abbreviation	Full form
AFM	Atomic Force Microscopy
ASTM	American Society for Testing and Materials
ATR	Attenuated Total Reflectance
BET	Brunauer–Emmett–Teller
CrI	Crystallinity Index
DSC	Differential Scanning Calorimetry
DTG	Derivative Thermogravimetry
EDAX	Energy-Dispersive X-ray Analysis
EDX	Energy-Dispersive X-ray Spectroscopy
FESEM	Field-Emission Scanning Electron Microscopy
FDM	Fused Deposition Modeling
FTIR	Fourier Transform Infrared Spectroscopy
FWHM	Full Width at Half Maximum
ICDD	International Centre for Diffraction Data
MCC	Microcrystalline Cellulose
NRC	Noise Reduction Coefficient
PLA	Poly(lactic acid)
SEM	Scanning Electron Microscopy
STL	Stereolithography
TGA	Thermogravimetric Analysis
UV–Vis	Ultraviolet–Visible Spectroscopy
WTL	Waste Tea Leaves
WTL-MCC	Waste-Tea-Leaf-Derived Microcrystalline Cellulose
XRD	X-ray Diffraction
α	Sound Absorption Coefficient
T_max_	Maximum Decomposition Temperature
R_a_	Average Surface Roughness
R_q_	Root-Mean-Square Surface Roughness
R_z_	Ten-Point Average Surface Roughness
S_a_	Areal Average Roughness
S_q_	Root-Mean-Square Areal Roughness
S_y_	Peak-to-Peak Surface Height
L/D	Screw Length-to-Diameter Ratio
RH	Relative Humidity
MFR	Melt Flow Rate
